# Rewiring of the FtsH regulatory network by a single nucleotide change in *saeS* of *Staphylococcus aureus*

**DOI:** 10.1038/s41598-017-08774-5

**Published:** 2017-08-16

**Authors:** Qian Liu, Mo Hu, Won-Sik Yeo, Lei He, Tianming Li, Yuanjun Zhu, Hongwei Meng, Yanan Wang, Hyunwoo Lee, Xiaoyun Liu, Min Li, Taeok Bae

**Affiliations:** 1 Department of Laboratory Medicine, Ren Ji Hospital, School of Medicine, Shanghai Jiao Tong University, Shanghai, 200127 China; 2 Institute of Analytical Chemistry and Synthetic and Functional Biomolecules Center, College of Chemistry and Molecular Engineering, Peking University, Beijing, 100871 China; 3 Department of Microbiology and Immunology, Indiana University School of Medicine-Northwest, Gary, Indiana, 46408 USA; 4 Department of Biopharmaceutical Sciences and Center for Biomolecular Sciences, College of Pharmacy, University of Illinois at Chicago, Chicago, Illinois 60607 USA; 5 Present Address: Johnson Medical Company Advanced Energy, Shanghai, 200233 China

**Keywords:** Pathogens, Infection

## Abstract

In the Gram-positive pathogen *Staphylococcus aureus*, the membrane-bound ATP-dependent metalloprotease FtsH plays a critical role in resistance to various stressors. However, the molecular mechanism of the FtsH functions is not known. Here, we identified core FtsH target proteins in *S*. *aureus*. In the strains Newman and USA300, the abundance of 33 proteins were altered in both strains, of which 11 were identified as core FtsH substrate protein candidates. In the strain Newman and some other *S*. *aureus* strains, the sensor histidine kinase SaeS has an L18P (T53C in *saeS*) substitution, which transformed the protein into an FtsH substrate. Due to the increase of SaeS L18P in the *ftsH* mutant, Eap, a *sae*-regulon protein, was also increased in abundance, causing the Newman-specific cell-aggregation phenotype. Regardless of the strain background, however, the *ftsH* mutants showed lower virulence and survival in a murine infection model. Our study illustrates the elasticity of the bacterial regulatory network, which can be rewired by a single substitution mutation.

## Introduction

In bacteria, ATP-dependent proteolysis machines (ClpXP, ClpAP, ClpCP, HslUV, Lon, and FtsH) play critical roles in protein quality control by eliminating misassembled or misfolded proteins and proteins no longer needed^1^. Of those proteases, FtsH is a Zn metalloprotease tethered to the cell membrane via the two transmembrane segments located at N-terminus^2^. The large cytoplasmic region of FtsH contains the ATPase and the protease domains essential for the substrate degradation. The ATPase domain contains the conserved Walker A (GXXXXGKT/S) and Walker B (hhhhDE, where h = hydrophobic amino acid) motifs, which are required for ATP binding and hydrolysis, while the protease domain contains the Zn-metalloprotease active site (HEXXH)^2–4^. FtsH exists as a homohexameric protein, where the ATPase domain forms a hexameric ring with an axial pore. The FtsH substrates are fed through the axial pore into the proteolytic chamber and degraded into small peptides^5^. Due to its weak unfoldase activity, FtsH cannot degrade tightly folded proteins and targets only unfolded or loosely folded proteins-either cytoplasmic or membrane-bound^6,7^.

To understand the molecular function of FtsH, it is imperative to identify FtsH substrate proteins. FtsH substrates have been identified mostly at an individual protein level by assessing the effect of *ftsH* mutation on the abundance of the protein being examined^8–10^. Two studies, however, attempted to identify FtsH substrates on a global scale. Using a protease-deficient FtsH mutant as a trap for FtsH substrates in *Escherichia coli*, Westphal *et al*. identified 15 proteins that were co-purified by the FtsH trap protein. Of the 15 proteins, one protein, LpxC, is a known FtsH substrate, and only four out of the remaining 14 proteins were shown to be degraded by FtsH, indicating a rather low sensitivity and specificity of the approach^11^. Lűdke *et al*., on the other hand, used anionic exchange chromatography/SDS-PAGE and two-dimensional gel electrophoresis to find 10 cytoplasmic and membrane proteins whose abundance is significantly increased in the *ftsH*-deletion mutant of *Corynebacterium glutamicum*^12^. However, this method requires rather extensive sample preparations and does not exclude the possibility that some of the proteins might be affected by FtsH indirectly.

*Staphylococcus aureus* is a Gram-positive pathogen, causing diverse diseases from skin and soft-tissue infections to life-threatening infections such as sepsis, pneumonia, endocarditis, and toxic shock syndrome^13^. In *S*. *aureus*, the *ftsH*-deletion mutant showed increased sensitivity to various stress conditions (e.g., high salt, amino acid deficiency, acid, and tellurite) and decreased survival in a murine skin infection model^14^. However, the underlying molecular mechanisms of those phenotypes have not yet been understood.

*S*. *aureus* strain Newman is a methicillin-sensitive clinical isolate of *S*. *aureus*^15^ whereas USA300 is the predominant methicillin-resistant *S*. *aureus* (MRSA) in the United States^16^. Intriguingly, in this study, we found that the deletion of *ftsH* causes cell-aggregation in Newman, but not in USA300. Cell aggregates are a multicellular structure in a planktonic state. Bacterial cells in the planktonic aggregates show increased metabolic activity and mutation rate as well as higher tolerance to antibiotics^17^ and resistance to phagocytosis^18^. To understand the molecular mechanism of the strain-specific cell aggregation by the *ftsH*-deletion, we determined the FtsH substrate proteins by mass spectrometry (MS)-based comparative proteomics in both strains and found that the L18P substitution mutation in the sensor histidine kinase of SaeS (T53C in *saeS*) is responsible for the strain-specific phenotype. Our study illustrates an example of how a single nucleotide change can rewire a bacterial regulatory network, conferring an individuality to the bacterial strain.

## Results

### Deletion of *ftsH* causes cell-aggregation in strain Newman, but not in USA300

**Figure 1:**
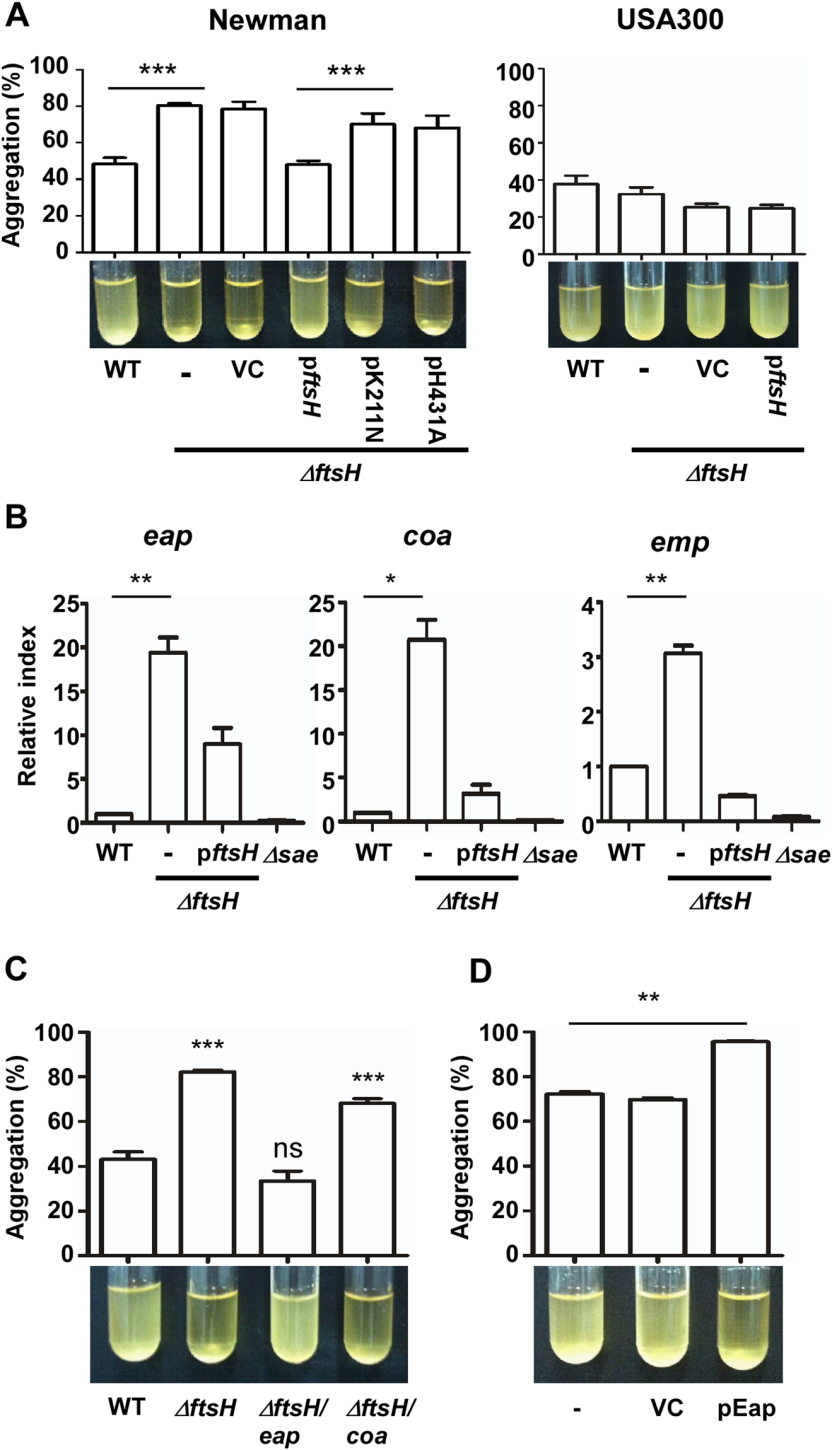
The *ftsH* deletion produces distinct phenotype in *S*. *aureus* Newman and USA300. (**A**) Effect of *ftsH*-deletion on the aggregation of *S*. *aureus* Newman and USA300. WT, wild type; Δ*ftsH*, deletion mutant of *ftsH*; -, no plasmid; VC, pCL55; p*ftsH*, pCL55 carrying a wild type copy of *ftsH*; pK211N, pCL55 carrying an *ftsH* gene with K211N mutation (*ftsH*^K211N^); pH431A, pCL55 carrying an *ftsH* gene with H431A mutation (*ftsH*^H431A^). ***p ≤ 0.001 by two-tailed Student’s t-test. (**B**) Effect of the *ftsH* deletion on the transcription of *eap*, *coa*, and *emp* in the strain Newman. The data are derived from three biological repeats of qRT-PCR analysis. Δ*sae*, the *sae*-deletion mutant. *p ≤ 0.05; **p ≤ 0.01 by two-tailed Student’s t-test. (**C**) The role of *eap* in the aggregation of Newman. The aggregations of the mutant strains were compared with that of wild type (WT). *eap*, transposon insertion mutation in *eap*; *coa*, transposon insertion mutation in *coa*. ns, not significant; ***p ≤ 0.001 by two-tailed Student’s t-test. (**D**) Effect of Eap overexpression on the aggregation of Newman. VC, pYJ335; pEap, pYJ335 carrying a wild type copy of *eap*. **p ≤ 0.01 by two-tailed Student’s t-test.

To study the roles of FtsH in two different strain backgrounds, we deleted the *ftsH* gene in *S*. *aureus* strain Newman and USA300. Intriguingly, the deletion enhanced cell-aggregation only in strain Newman (WT vs – in Fig. 1A). Introduction of wild type *ftsH* gene reduced the cell-aggregation (p*ftsH* under Newman in Fig. 1A), whereas the *ftsH* genes carrying a mutation either in the Walker A box (K211N) or in the metalloprotease active site (H431A) failed to do so, demonstrating that the function of FtsH, not the mere presence of the protein, is required to limit the cell-aggregation. These results also suggest that FtsH plays distinct roles in two different strain backgrounds.

### Identification of FtsH substrate protein candidates in Newman and USA300

**Figure 2:**
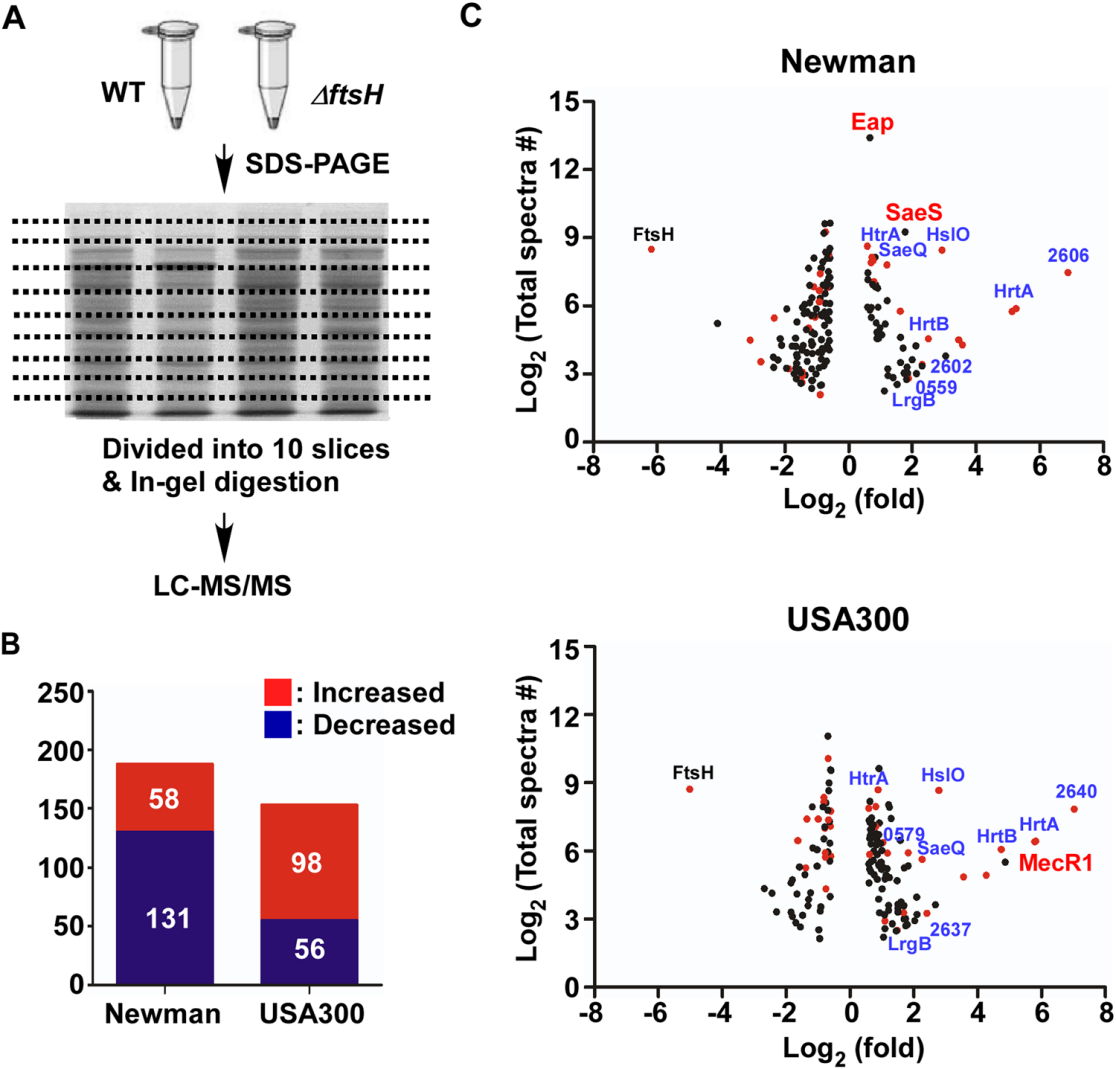
Comparative proteomic profiling of the *ftsH* mutant of *S*. *aureus* Newman and USA300. (**A**) A schematic diagram of the proteomic workflow for proteomic profiling of the wild type and the *ftsH* deletion mutant (Δ*ftsH*) of *S*. *aureus* Newman and USA300. (**B**) Summary of proteins whose abundance was significantly changed in the *ftsH* mutant. Increased, abundance increased in Δ*ftsH*; Decreased, abundance decreased in Δ*ftsH*. (**C**) Distribution of staphylococcal proteins significantly altered in the *ftsH* mutant of the strains Newman and USA300. In total, 1977 proteins were identified in *S*. *aureus* Newman, and 2056 proteins in USA300. For clarity, only the proteins altered 1.5 times or more were plotted. The X-axis represents the average fold changes of proteins (Δ*ftsH*/WT) while the Y-axis shows the abundance (i.e., total spectral counts) of the corresponding proteins. Red dots indicate the proteins altered in both strains while black dots are the proteins altered only in one strain. Names of the core FtsH substrate candidates are shown in blue while the names of the strain-specific substrates are shown in red.

To investigate the molecular mechanism underlying the strain-specific cell-aggregation, we decided to determine the proteins whose abundance is affected by the *ftsH* deletion. We collected cells at early stationary growth phase (Supplementary Fig. 1), where the bacteria are expected to experience various environmental stressors such as depletion of nutrients and accumulation of harmful metabolites. After cell lysis, total proteins were separated by SDS-PAGE; then the gel was divided into 10 pieces. The proteins in each gel slice were digested by trypsin and analyzed by LC-MS/MS (Fig. 2A). Abundance of 189 and 154 proteins was significantly altered in the *ftsH* deletion mutant of Newman and USA300, respectively (Fig. 2B,C and Supplementary Tables 1–4). Intriguingly, only 33 proteins (~ 20%, 17 increased, 16 decreased) were commonly altered in both strains (red dots in Fig. 2C and Table 1), indicating that the effect of *ftsH* deletion is indeed very distinct in those strains. For example, in Newman, 131 proteins were decreased in their abundance by the *ftsH* deletion while, in USA300, only 56 proteins were decreased (Fig. 2B and Supplementary Tables 2 and 4). The *ftsH* deletion affected proteins of diverse cellular functions including multiple proteins of unknown functions (Supplementary Fig. 2). These results suggest that FtsH plays diverse and heterogeneous roles in different strains.

**Table 1:** Proteins commonly affected by the *ftsH* deletion in Newman and USA300.

NM	USA	Gene	Product	Fold changes (ftsH/WT)^**a**^		
				Mass Spec		RNA-seq
**Up**-**regulated** (**17**)				**NM**	**USA**	**USA**
**0197**^**c**^	**0257**	***lrgB***	**antiholin**-**like protein LrgB**	**3**.**6**	**2**.**8**	**0**.**5**
**0252**	**0310**		**hypothetical protein**	**3**.**1**	**3**.**6**	- ^**b**^
0474	0490	*hslO*	Hsp33-like chaperonin	7.7	6.9	7.2
**0559**	**0579**		**hypothetical protein**	**3**.**7**	**2**.**1**	—
**0676**	**0692**	***saeQ***	**SaeS regulatory protein SaeQ**^**d**^	**1**.**6**	**4**.**8**	—
**0887**	**0918**	***murG***	**diacylglycerol glucosyltransferase**	**11**.**0**	**11**.**8**	—
**0952**	**0986**	***cydA***	**cytochrome D ubiquinol oxidase**, **subunit I**	**1**.**7**	**2**.**3**	—
**0981**	**1016**	***cyoE***	**protoheme IX farnesyltransferase**	**12**.**0**	**19**.**3**	—
**1147**	**1130**	***ffh***	**signal recognition particle protein**	**1**.**6**	**1**.**5**	—
**1371**	**1351**		**hypothetical protein**	**2**.**3**	**1**.**8**	—
**1566**	**1619**	***hemA***	**glutamyl**-**tRNA reductase**	**38**.**2**	**56**.**4**	—
**1621**	**1674**	***htrA***	**putative serine protease**	**1**.**7**	**1**.**9**	—
2261	2306	*hrtA*	ATP-binding protein	35	55.3	127.0
2262	2307	*hrtB*	ABC transporter permease protein	5.7	26.8	211.0
2529	2565	*clfB*	clumping factor B precursor	1.5	1.8	1.5
2602	2637		conserved hypothetical protein	5.0	5.3	14.2
2606	2640		putative transcriptional regulator	117.4	130.7	365.9
**Down**-**regulated** (**16**)						
0136	0194	*ptsG*	sucrose-specific PTS transporter IIBC	0.4	0.6	—
0473	0489	*ftsH*	ATP-dependent metalloprotease	0.0	0.0	0.0
0885	0916		hypothetical protein	0.6	0.7	—
0933	0966	*purE*	phosphoribosylaminoimidazole carboxylase catalytic subunit	0.5	0.5	—
0948	0982		hypothetical protein	0.6	0.6	1.4
1162	1145	*xerC*	tyrosine recombinase xerC	0.4	0.6	—
1459	1516		ATP-binding protein	0.4	0.7	—
1603	1655	*ald*	alanine dehydrogenase	0.5	0.6	1.6
1681	1731	*pckA*	phosphoenolpyruvate carboxykinase	0.6	0.6	—
1847	1890	*sspB*	staphopain thiol proteinase	0.2	0.6	—
1922	1970		phage exonuclease	0.5	0.7	—
2093	2149	*lacG*	6-phospho-beta-galactosidase	0.2	0.3	—
2096	2152	*lacD*	tagatose 1,6-diphosphate aldolase	0.1	0.4	—
2232	2278	*hutU*	urocanate hydratase	0.3	0.6	—
2509	2545	*betA*	choline dehydrogenase	0.5	0.4	—
2510	2546	*betB*	glycine betaine aldehyde dehydrogenase	0.5	0.6	—
**Differentially regulated** (**3**)						
1242	1228	*thrB*	Homoserine kinase	0.4	2.1	0.5
1579	1632	*nrdR*	Transcription regulator NrdR	0.5	1.5	—
2523	2558	*nsaS/braS*	Histidine kinase	0.5	3.2	—

^a^Listed are the results with p ≤ 0.05 by Student’s t-test (two-tailed, paired). Statistical analysis results can be found in S1-5Tables; ^b^ -, no significant change; ^c^The 11 putative FtsH substrate proteins were bold-faced; ^d^The four proteins whose degradation by FtsH was demonstrated *in vitro* (Fig. 4C) were underlined.

### The effect of the *ftsH* deletion on transcriptome of the strain USA300

**Figure 3:**
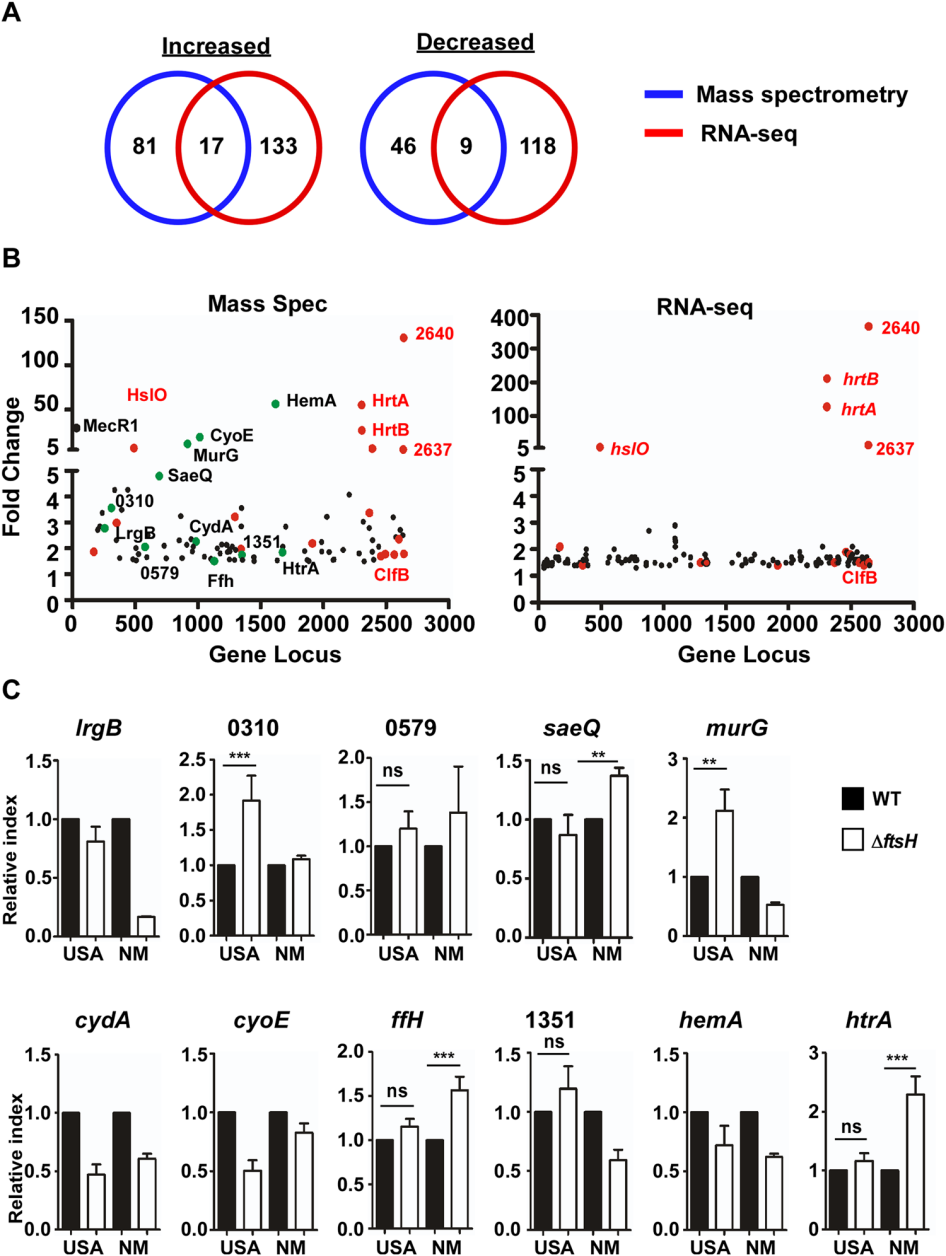
Identification of the potential core FtsH substrates in *S*. *aureus*. (**A**) Summary of the proteome and transcriptome analyses of USA300Δ*ftsH* (fold change ≥1.5; p ≤ 0.05 by two-tailed paired Student’s t-test). (**B**) Comparison of the proteome (left panel) and transcriptome (right panel) analyses results for upregulated proteins and genes. Red dots indicate the 17 up-regulated proteins and genes shown in Fig. 3A. The green dots are the potential core FtsH substrate proteins. (**C**) Confirmation of the RNA-seq results by qRT-PCR for the 11 genes encoding the putative core FtsH substrates. Gene name or gene identifier of the genome of USA300_FPR3757 is shown above each graph. WT, wild type; Δ*ftsH*, *ftsH* deletion mutant; USA, USA300; NM, Newman.

To assess the impact of the *ftsH* deletion on the transcriptional profile in *S*. *aureus*, we carried out RNA-seq analysis for the wild type and the *ftsH* deletion mutant of USA300. A total of 277 genes (150 up-regulated, 127 down-regulated) were transcriptionally affected by the *ftsH* deletion (Supplementary Table 5). Surprisingly, only 26 genes (17 up-regulated and 9 down-regulated) showed a correlation between protein and transcript levels (Fig. 3A,B, Supplementary Table 6, and Supplementary Fig. 3), implying that, in *S*. *aureus*, FtsH might have a broad impact on the post-transcriptional regulation.

### Identification of the potential core FtsH substrates in *S*. *aureus*

For identification of the potential FtsH substrate proteins, we set out three criteria: First, the substrate proteins should be either a cytoplasmic protein or a membrane protein; second, substrate proteins should be more abundant in the *ftsH* deletion mutant in both strain backgrounds; and third, the transcription of FtsH substrate gene should not be significantly up-regulated by the *ftsH* deletion at least in one of the strains. By comparing the proteins whose abundance was increased by the *ftsH* deletion, we identified 17 candidate proteins for FtsH substrate (Table 1). Among the proteins, we excluded ClfB because, as a cell surface protein anchored to cell wall via the LPXTG motif^19^, it is unlikely that the protein is targeted by FtsH. Of the remaining 16 proteins, the transcript levels of the following five proteins were elevated in USA300Δ*ftsH*: HslO, HrtA, HrtB, SAUSA300_2637, and SAUSA300_2640 (Table 1 and Supplementary Table 5). qRT-PCR analysis further confirmed that transcripts of those five proteins were elevated in the Δ*ftsH*-deletion mutant of Newman (NMΔ*ftsH*) too (Supplementary Fig. 4), strongly suggesting that those proteins are likely affected by FtsH indirectly at a transcriptional level. On the other hand, qRT-PCR analysis showed that the transcription of the remaining 11 proteins was not increased at least in one of the strains (Fig. 3C), indicating that the following 11 proteins are possibly the core FtsH substrate proteins in *S*. *aureus*: LrgB, SAUSA300_0310, SAUSA300_0579, SaeQ, MurG, CydA, CyoE (CyoD in Newman), Ffh, SAUSA300_1351, HemA, and HtrA (Table 1).

### Confirmation of select FtsH substrate proteins

**Figure 4:**
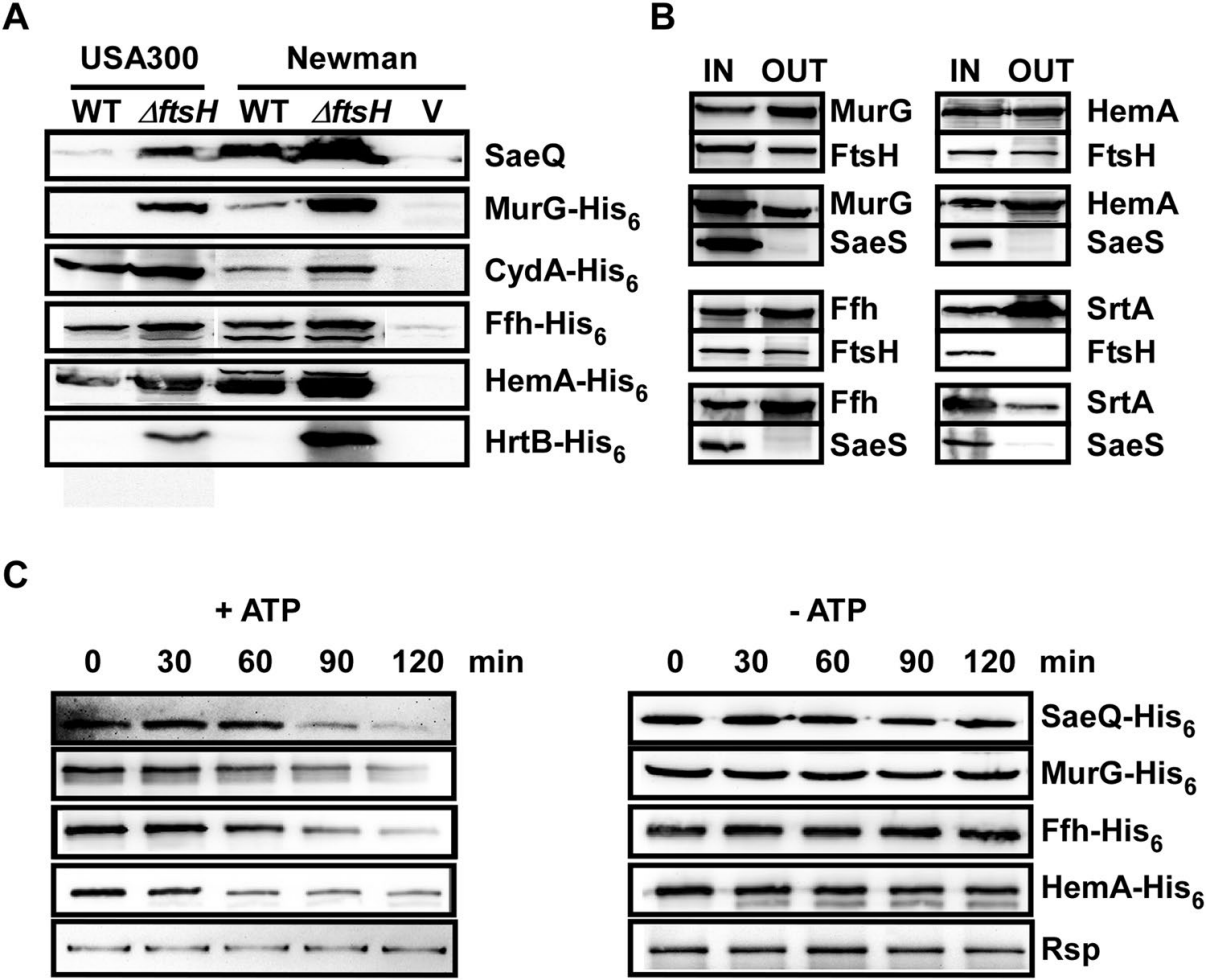
Confirmation of select FtsH substrate proteins. (**A**) Western blot analysis of six FtsH substrate proteins. An equal amount of cells was used for the analysis (see Methods). SaeQ was detected by anti-SaeQ antibody while other proteins were by anti-His_6_-tag antibody. WT, wild type; Δ*ftsH*, *ftsH*-deletion mutant; V, a *sae* mutant of Newman (for SaeQ) or vector (i.e., pYJ335) control (for other proteins). The full-length blots and the quantification results are presented in Supplementary Fig. 5. Since, for some proteins, Western blot was carried out for USA300 and Newman on a different gel, only the comparison between WT and Δ*ftsH* of the same strain are valid. (**B**) Co-purification assay to examine the interaction between FtsH and substrate proteins. From cell lysate, select substrate proteins (MurG-His_6_, Ffh-His_6_, and HemA-His_6_) and SrtA-His_6_ were purified with Ni-agarose; then FtsH^H431A^-Flag and SaeS-Flag were detected by Flag-tag antibody. SaeS and SrtA are not an FtsH substrate and used as negative controls. For clarity, His_6_ and Flag-tag names were omitted in the protein names. IN, input sample; OUT, proteins purified by Ni-column chromatography. The full-length blots are presented in Supplementary Fig. 6. (**C**) Proteolytic degradation of four substrate proteins by FtsH. Membrane vesicles containing FtsH were used as a source for FtsH while either membrane vesicles containing SaeQ (SaeQ) or purified proteins (MurG, Ffh, and HemA) were used as a substrate. Rsp, a non-substrate protein was used as a control. Proteins were detected by Western blot analysis with anti-His_6_ antibody. The quantification results are presented in Supplementary Fig. 7C.

To confirm the proteome analysis results, we decided to compare the protein level of the six proteins, SaeQ, MurG, CydA, Ffh, HemA, and HrtB in wild type and Δ*ftsH* strains. Except for SaeQ, which was expressed from the chromosome, all other substrate proteins were expressed from the multi-copy plasmid pYJ335 as a C-terminal His-tagged protein. Indeed, the abundance of all of the substrate proteins was significantly increased in Δ*ftsH* in both strain backgrounds (Fig. 4A and Supplementary Fig. 5).

We also investigated whether the substrate proteins directly interact with FtsH by a co-purification method by using FtsH^H431A^, a protease-deficient mutant. Flag-tagged FtsH^H431A^ was expressed in NMΔ*ftsH* with one of the three His-tagged substrate proteins, MurG-His_6_, Ffh-His_6_, or HemA-His_6_. Then the substrate proteins were purified by Ni-column chromatography, and co-purification of either Flag-FtsH^H431A^ or the negative control SaeS was examined by Western blot analysis. A significant level of FtsH^H431A^ was co-purified with all three substrate proteins, whereas the negative control protein SaeS was not (Fig. 4B and Supplementary Fig. 6). When SrtA-His_6_, a non-substrate protein, was purified by Ni-column chromatography, FtsH^H431A^ was not detected (the right second bottom picture in Fig. 4B). Based on these results, we concluded that the test substrate proteins interact with FtsH in the *in vivo* condition.

To determine whether FtsH directly degrades its substrate proteins, we decided to carry out the proteolysis assay with FtsH and select substrate proteins. Since the cytoplasmic domain of FtsH (FtsHc Δ1–150) or GST-FtsHc, a fusion protein of glutathione S-transferase and FtsHc, showed either no or very low proteolytic activity (Supplementary Fig. 7A & B), we used membrane vesicles purified from an FtsH-overexpression strain as a source for FtsH. For substrate proteins, we tested one membrane protein (i.e., SaeQ) and three cytoplasmic proteins (MurG, Ffh, and HemA). Significant proteolysis was observed only for the substrate proteins in the presence of ATP (Fig. 4C and Supplementary Fig. 7C). No proteolysis of the substrate proteins was observed in the absence of ATP, indicating that the membrane vesicles do not have non-specific proteolytic activity toward the substrate proteins. In addition, regardless of the presence of ATP, Rsp, a non-FtsH substrate, was not degraded, strongly suggesting that FtsH degrades the substrate proteins in a specific manner.

Finally, the four substrate proteins (MurG, CydA, Ffh, and HemA) showed a significantly (2.4–7 fold) increased half-life (Supplementary Fig. 8), whereas the non-substrate protein SaeS showed only 1.5 times increase (SaeS^L^ in Supplementary Fig. 8). These results indicate that stabilization of the substrate proteins, not enhanced translation, is responsible for the elevated levels of the substrate proteins in the *ftsH*-deletion mutant.

### In strain Newman, the L18P mutation transforms SaeS into an FtsH substrate

**Figure 5:**
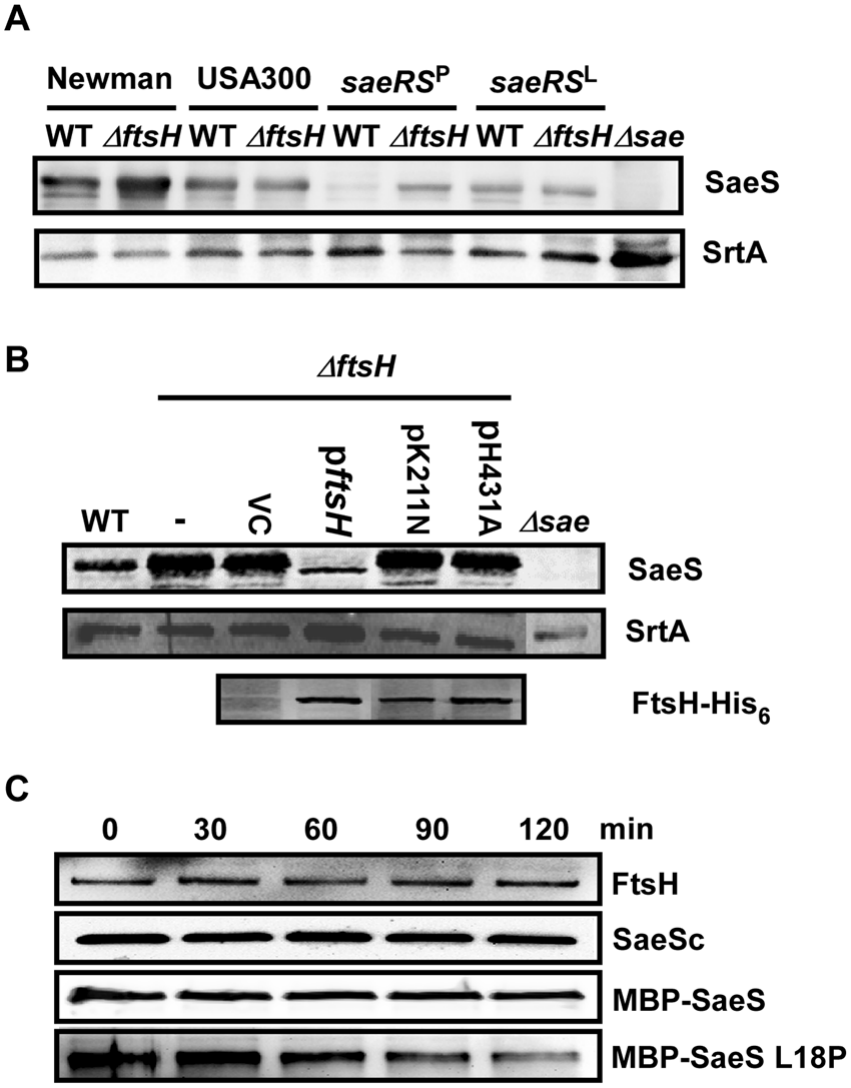
In strain Newman, the L18P mutation transforms SaeS into an FtsH substrate. (**A**) Effect of the *ftsH* deletion on the protein level of SaeS L18P. Cells were grown in TSB; then SaeS was detected by Western blot analysis. WT, wild type; Δ*ftsH*, *ftsH* deletion mutant; *saeRS*^P^, the *sae*-deletion mutant of Newman expressing SaeR and SaeS L18P (i.e., NMΔ*sae*[pCL-RS^P^]); *saeRS*^L^, the *sae*-deletion mutant of Newman expressing SaeR and the wild type SaeS (i.e., NMΔ*sae*[pCL-RS^L^]); Δ*sae*, the *sae*-deletion mutant of Newman. Sortase A (SrtA), a membrane protein, was used as a loading control. The full-length blots are presented in Supplementary Fig. 9A. (**B**) Role of the FtsH enzymatic activities on the degradation of SaeS L18P. Proteins were detected by Western blot analysis. To detect the FtsH protein expressed from the complementation plasmids, anti-His_6_-tag antibody was used. Sortase A (SrtA) was used as a loading control. WT, wild type Newman; Δ*ftsH*, the *ftsH*-deletion mutant of Newman; -, no plasmid; VC, the vector control pCL55; p*ftsH*, pCL55 carrying the wild type copy of *ftsH*; pK211N, pCL55 carrying the *ftsH* gene with K211N mutation at the ATPase domain; pH431A, pCL55 carrying the *ftsH* gene with H431A mutation at the protease domain; Δ*sae*, the *sae*-deletion mutant of Newman. The full-length blots are presented in Supplementary Fig. 9B. (**C**) Proteolysis of various forms of SaeS proteins by membrane vesicles containing FtsH. The membrane vesicles were mixed with the purified SaeS proteins; then the proteolysis was initiated by addition of ATP. Quantification results are presented in Supplementary Fig. 9C.

In the strain Newman, the abundance of two proteins, SaeS and Eap (extracellular adherence protein), was uniquely increased by the *ftsH*-deletion (Fig. 2C). As the sensor histidine kinase of the SaeRS two component system (TCS), SaeS is a membrane protein, while Eap is a secreted adhesin, a protein unlikely to be targeted by FtsH^20–22^. In the strain Newman and some other *S*. *aureus* strains such as LysK 2010 and 1801-1 2010, SaeS contains L18P (T53C in *saeS*) substitution mutation in the first transmembrane segment, resulting in constitutively high kinase activity^23–25^. The unique increase of SaeS in NMΔ*ftsH* indicates that the L18P mutation rendered SaeS susceptible to the proteolysis by FtsH, which was confirmed by Western blot analysis (Newman vs USA300 in Fig. 5A). Previously we showed that SaeS L18P is unstable, and that SaeQ protects SaeS L18P from proteolytic degradation^26^. Indeed, without SaeQ, almost no SaeS L18P was detected in the presence of FtsH (WT under *saeRS*^*P*^ in Fig. 5A); however, when the *ftsH* gene was deleted, the abundance of SaeS L18P was increased to a detectable level (*ΔftsH* under *saeRS*^*P*^ in Fig. 5A). In contrast, the abundance of the wild type SaeS was not affected either by SaeQ or by FtsH (USA300 and *saeRS*^*L*^ in Fig. 5A). The introduction of the wild type *ftsH* gene reduced the abundance of SaeS L18P, whereas the mutant *ftsH* genes failed to do so (Fig. 5B). When the FtsH-containing membrane vesicles were mixed with the cytoplasmic domain of SaeS (SaeSc), maltose binding protein (MBP)-SaeS fusion, or MBP-SaeS L18P fusion in the presence of ATP, only MBP-SaeS L18P was significantly degraded (Fig. 5C and Supplementary Fig. 9C)_._ Finally, the deletion of *ftsH* increased the half-life of SaeS L18P by approximately 10 times (from 23 min to 224 min) whereas the half-life of the wild type SaeS was slightly increased from 31 min to 47 min (Supplementary Fig. 8). These results demonstrate that, in the strain Newman, the L18P mutation (i.e., the T53C single nucleotide change in *saeS*) transformed the SaeS protein into an FtsH substrate and placed the SaeRS TCS under the regulatory control of FtsH.

### The increased abundance of Eap is responsible for the enhanced cell-aggregation of NMΔ*ftsH*

In NMΔ*ftsH*, along with SaeS L18P, Eap was also specifically increased in abundance (Fig. 2A). As an adhesin with affinity to various host molecules such as fibronectin, fibrinogen, and prothrombin, Eap is known to mediate cell-aggregation in *S*. *aureus*^22^. In addition, as a member of *sae*-regulon, the transcription of *eap* is activated by the SaeRS TCS^27^. Indeed, the transcription of *eap* was increased in the *ftsH* mutant, along with other members of *sae* regulon, *coa* and *emp*, whereas the deletion of *sae* abolished their transcriptions (Fig. 1B). By contrast, the transcription of those genes was not affected by the *ftsH* deletion in the USA300 strain background (Supplementary Fig. 10). These results indicate that, in NMΔ*ftsH*, the abundance of Eap was increased due to the elevated level of SaeS L18P.

To examine whether Eap is responsible for the enhanced cell-aggregation of NM*ΔftsH*, we disrupted the *eap* gene in NMΔ*ftsH* and assessed the effect on cell-aggregation. As shown in Fig. 1C, the disruption of *eap* reduced the self-aggregation of the NMΔ*ftsH* to the wild type level, whereas the disruption of the *coa* gene did not. In addition, the introduction of a multi-copy plasmid carrying the *eap* gene increased the cell-aggregation of the wild type Newman cells (pEap in Fig. 1D). Based on these results we concluded that, in the strain Newman, the single nucleotide change T53C in *saeS* linked the two independent regulatory systems, the SaeRS TCS and FtsH, causing the strain-specific aggregation of the *ftsH*-deletion mutant.

### Regardless of the state of the SaeRS TCS, FtsH is critical for survival of *S*. *aureus* during systemic infection

**Figure 6:**
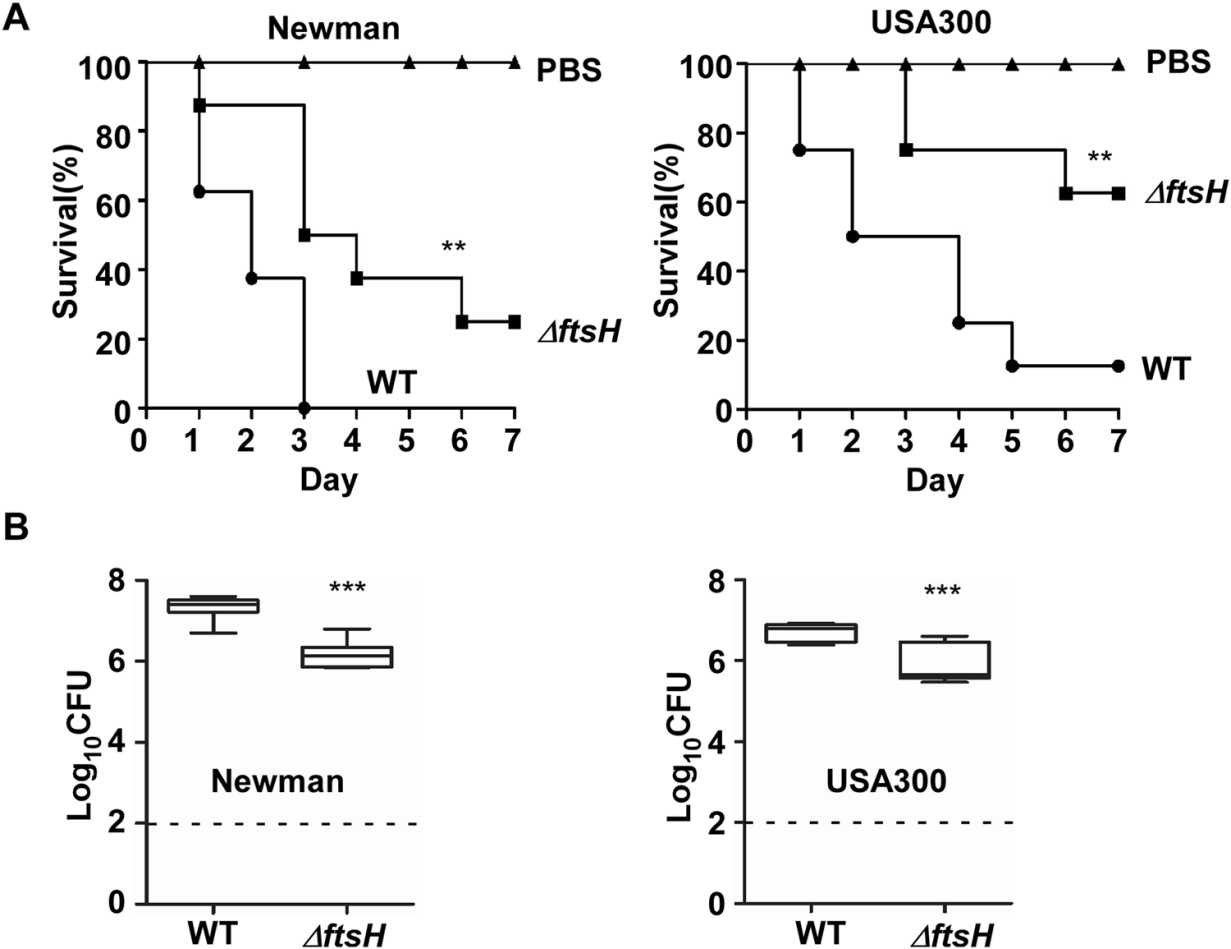
FtsH is critical for *in vivo* survival of *S*. *aureus* during infection. (**A**) The role of FtsH in staphylococcal virulence during murine infection. The test strains were grown in TSB until exponential growth phase; then test strains were administered into eight mice via retro-orbital route. The significance of the survival was analyzed by Log-rank (Mantel-Cox) test. **p ≤ 0.01.The experiment was repeated, and similar results were observed. (**B**) The role of FtsH in the survival/growth of *S*. *aureus* during murine infection. At day 4 post infection, eight mice were killed, and the kidneys were harvested and ground. The colony forming unit (CFU) of *S*. *aureus* in the ground kidneys was measured by serial dilution method on tryptic soy agar. The detection limit is indicated by a dashed line.***p ≤ 0.001 by two-tailed Student’s t-test.

A previous study showed that disruption of *ftsH* in *S*. *aureus* SH1000 reduced staphylococcal survival in a murine skin infection model^14^. On the other hand, the aggregation might impose a protective effect on NMΔ*ftsH*. In addition, the SaeRS TCS controls more than 20 important virulence factors and is critical for *in vivo* survival of *S*. *aureus*^21,28,29^. In particular, the kinase activity of SaeS correlated well with staphylococcal virulence in a murine infection model^30^. Since, in NMΔ*ftsH*, the absence of FtsH brought about bacterial aggregation and higher activity of SaeRS TCS, we asked the question whether the bacterial aggregation and the heightened Sae activity can compensate for the absence of FtsH. Regardless of the strain background, the *ftsH* mutant showed reduced virulence (Fig. 6A) and lower numbers of bacteria in kidney (Fig. 6B), showing that FtsH contributes to *in vivo* survival and/or growth of *S*. *aureus* in the kidney, regardless of its effect on cell aggregation and the SaeRS TCS.

## Discussion

In this study, by combining MS-based proteomics and RNA-seq, we identified 11 potential FtsH core substrate proteins in *S*. *aureus* (Fig. 2 and Table 1). Surprisingly, the Newman-specific aggregation of the Δ*ftsH*-deletion mutant was traced to the T53C mutation in *saeS*, which not only increases the kinase activity of the SaeS protein, but also transforms the SaeS protein into an FtsH substrate, linking the two regulatory systems together (Fig. 5). Our study illustrates an example of how a single nucleotide change can rewire bacterial regulatory network and confer individuality to the bacterial strain.

As compared with previous approaches, our MS-based method is rather straightforward, requiring only the SDS-PAGE analysis of wild type and *ftsH* mutant cell lysates before MS analysis. This quantitative proteomic workflow provides high throughput, robust quantification, and much higher coverage of proteome than two-dimensional electrophoresis-based methods. Although the MS-based approach cannot distinguish substrate proteins from the proteins affected by FtsH indirectly, the highly distinct effects of *ftsH* deletion on the proteome profile in two different strains allowed us to identify 17 proteins whose abundance was increased in both strains. Of the 17 proteins, five proteins were excluded from the list of potential FtsH substrates due to their transcriptional up-regulation in both strains (Table 1 and Supplementary Fig. 4). However, because the transcriptional regulation and degradation by FtsH are not mutually exclusive, we cannot formally rule out the possibility that some of the excluded proteins are a genuine FtsH substrate. In addition, since we used only one biological sample for each strain at one growth condition (i.e., early stationary growth phase in a rich medium), it is possible that our proteomic analysis did not detect all FtsH substrate proteins, and that different sets of FtsH substrates might emerge in different growth conditions.

One of the surprising findings in our study is the highly strain-specific effect of the *ftsH*-deletion in the strain Newman and USA300. Of the proteins affected by the *ftsH*-deletion (i.e., 189 and 154 in the strain Newman and USA300, respectively), approximately 80% of the proteins (i.e., 155 in Newman and 120 in USA300) were identified only in one strain. This is in line with recent studies showing that the profiles of surface protein and secreted protein are very distinct among different strains of *S*. *aureus*^31,32^. The heterogeneous nature of those proteomic profiles could be due to different genome content and disparate gene regulations^32^. In our study, both Newman and USA300 belong to multilocus sequence type 8 (MLST-8) and have similar genome sequences. In fact, of the proteins affected by the *ftsH* deletion, only 3–7% (5 out of 154 proteins in USA300 and 13 out of 189 proteins in Newman) were uniquely present in each strain (Supplementary Tables 1–4). Therefore, the disparate gene regulation is likely the major cause of the heterogeneity observed in this study. Indeed, uniquely in Newman, the SaeRS TCS is negatively regulated by FtsH via SaeS degradation (Figs 2C and 5). In addition, transcription regulators NrdR and the histidine sensor kinase NsaS/BraS were affected by the *ftsH* deletion in an opposite manner (Table 1), suggesting that the proteins under the control of those regulatory systems are likely affected differentially by *ftsH* deletion. The molecular mechanism and the physiological significance of the distinct effect on those transcription regulators, however, remain to be determined.

One interesting observation in our study is that, despite their sequence homology (60–70%), FtsH proteins show very different substrate repertoires among different bacteria. In *E*. *coli*, FtsH is known to degrade 15 proteins (i.e, ATPase F_0_α, DadA, FdoH, IscS, KdtA, λCII, λCIII, λXis, LpxC, PspC, RpoH, SecY, SoxS, YccA, YfgM) along with SrrA-tagged proteins^9,11^. In *Corynebacterium glutamicum*, FtsH was predicted to digest at least nine proteins (i.e., AccBC, AceA, AceB, Gap, MetE, NCgl1985, SdhA, SdhB, SdhCD)^12^. However, none of orthologous proteins was identified in our study (Table 1) and, in fact, there is no single FtsH substrate protein shared by all three bacteria. These results may indicate that FtsH proteins of different bacteria have adapted to target different sets of substrate proteins. To a certain extent, disparate protein expressions can explain the distinct FtsH substrate repertoires. For example, among the 15 FtsH substrate proteins in *E*. *coli*, only two proteins, ATPase F_0_α and SecY, exist in *S*. *aureus*. It is possible that bacteria-specific regulators or adaptors are involved in FtsH substrate selection in each bacterium. In *E*. *coli*, YccA, QmcA, and HflKC are shown to bind to FtsH and modulate the substrate degradation of FtsH^33–35^. In addition, the endopeptidase HtpX contributes to the FtsH-mediated membrane protein degradation by cleavage of the cytoplasmic loop in the substrate proteins^36–38^. In *Salmonella enterica*, the binding of MgtR, a hydrophobic membrane peptide, to the membrane protein MgtC promotes the degradation of MgtC by FtsH^39^. Therefore, the FtsH substrate selection seems to be determined not only by the inherent enzymatic characteristics of FtsH, but also by other factors such as regulators, collaborators, and adaptors. Neither of the regulatory molecules described above is found in *S*. *aureus*, and it remains to be determined whether regulatory molecules similar to those found in *E*. *coli* or *S*. *enterica* are involved in the FtsH substrate selection in *S*. *aureus*.

In a previous study, Lithgow *et al*. showed that the *ftsH* mutant of *S*. *aureus* shows pleiotropic defects such as slow growth, reduced osmotolerance, increased sensitivity to multiple unrelated stressors (e.g., acid, methyl viologen, and potassium tellurite), and reduced survival during starvation of amino acid and phosphate^14^. In this study, we identified 33 proteins commonly affected in the strains Newman and USA300 (Table 1). Of the 33 proteins, MurG is involved in cell wall synthesis, HemA and CyoE in heme biosynthesis, and PurE in nucleotide synthesis. Perturbation of the abundance of those proteins might negatively affect the overall function of those biosynthesis pathways, resulting in slow growth and lower fitness during infection. In *S*. *aureus*, along with proline, glycine betaine is a major osmoprotectant^40^. The deletion of *ftsH* lowered the abundance of two enzymes involved in the synthesis of the osmoprotectant (*betA* and *betB* in Table 1), which might explain the reduced osmotolerance of the *ftsH* mutant^14^. Finally, of the 17 proteins whose abundance was increased in the *ftsH* deletion mutant, the following 10 are predicted to be a membrane protein: LrgB, SAUSA300_0310, SAUSA300_0579, SaeQ, CydA, CyoE, SAUSA300_ 1351, HtrA, HrtB, and SAUSA300_2640. Therefore, the increased abundance of those proteins in the cell membrane might impose stresses to the cell membrane in a non-specific manner, resulting in the pleotropic sensitivity to various stressors. Certainly, more work is needed to define the exact molecular mechanism by which FtsH plays its critical role in those cellular functions.

## Methods

### Bacterial strains, plasmids and culture conditions

The bacterial strains and plasmids used in this study are listed in Supplementary Table 7. *E*. *coli* and *S*. *aureus* were grown in Luria-Bertani broth and tryptic soy broth (TSB), respectively. For transduction of mutations and plasmids, heart infusion broth (HIB) supplemented with 5 mM CaCl_2_ was used. When necessary, antibiotics were added to the growth media at the following concentrations: ampicillin, 100 μg mL^−1^; erythromycin, 10 μg mL^−1^; and chloramphenicol, 5 μg mL^−1^.

### DNA manipulation

Unless stated otherwise, all restriction enzymes and DNA modification enzymes were purchased from New England Biolabs. Plasmids and genomic DNA were extracted with plasmid miniprep kit (Zymo research). Plasmid DNA was introduced into *E*. *coli* by the method of Hanahan^41^ and into *S*. *aureus* RN4220 by electroporation with a gene pulser (Bio-Rad).

### Generation of mutant and complementation strains

We used a ligation independent cloning (LIC) method to delete *ftsH*^42^. Vector DNA was PCR-amplified from pKOR1 using the primers P236/237 (Supplementary Table 8). Two 1 kb DNA fragments, upstream and downstream of *ftsH*, were PCR-amplified from the chromosomal DNA with the primer pairs P83/84 and P85/86 (Supplementary Table 8) and PrimeSTAR^TM^ (Takara). The PCR products were treated with T4 DNA polymerase in the presence of dCTP (vector) or dGTP (insert DNA) and mixed together. The DNA mixture was transformed into *E*. *coli* DH5α. The resulting plasmid, pKOR1Δ*ftsH* was electroporated into *S*. *aureus* strain RN4220 and subsequently transduced into Newman and USA300 with ϕ85. The *ftsH* deletion was carried out as described previously^43^.

To generate *ftsH* complementation plasmid, vector DNA was PCR-amplified from pCL55^44^ with the primers P35/80, while the *ftsH* gene with its own promoter was amplified with the primers P2507/2508 (for His-tag at C-terminus) or P2507/PL47 (for Flag-tag at C-terminus) (Supplementary Table 8). The plasmid was assembled with LIC as described above.

The complementation plasmids carrying mutant *ftsH* genes were generated by site-directed mutagenesis as described by Ho *et al*.^45^ with the following primers: P2576, P2577, P2578, and p2579 (Supplementary Table 8). The mutations were verified by DNA sequencing.

To generate *eap* and *coa* mutants, transposon insertion mutations were transduced from the *Phoenix* library mutant strains to the strain Newman or NMΔ*ftsH* with ϕ85.

To construct the overexpression plasmid for *eap* (pEap), the *eap* gene was PCR-amplified with primers P319/P320 (Supplementary Table 8). The amplified fragment was digested with *Xho*I/*Kpn*I and inserted into pYJ335.

### Proteomic profiling of WT and *ftsH* mutant strains

Overnight culture in TSB was diluted 100 times with TSB and incubated at 37 °C for 8 h. Cells were collected by centrifugation and treated with lysostaphin (50 μg mL^−1^) at 37 °C for 30 min. Bacterial cell lysates were separated by 10% SDS-PAGE and stopped when the dye front reached 2 cm below the stacking gel. The gel was divided equally into ten slices and subjected to in-gel protein digestion^46^; then the extracted peptide samples were analyzed by LC-MS/MS on a nanoflow liquid chromatography (EASY-nLC1000, Thermo Scientific) coupled with a linear ion trap mass spectrometer (LTQ Velos Pro, Thermo Scientific) in a data-dependent mode. Triplicate LC-MS/MS runs were conducted for each sample, thereby allowing the assessment of the technical reproducibility. Detailed procedures of in-gel digestion and LC-MS/MS settings were carried out as described previously by Hu *et al*.^47^.

### Identification of proteins and quantitative analysis of their relative abundance

Peptides and proteins were assigned by a database searching method. LC-MS/MS raw files were searched with MASCOT 2.3.02 (Matrix Science, London, UK) against *S*. *aureus* protein database of strains Newman or USA300 from NCBI (http://www.ncbi.nlm.nih.gov/protein/). The resulting peptide and protein assignments were filtered to achieve a peptide false discovery rate (FDR) of 1% using the target-decoy method. Relative protein abundance between different samples was assessed using a spectral counting method^48^. Spectral counts represent the total number of repeated identification of peptides for a given protein during the entire analysis and provide a semi-quantitative measurement of protein abundance. Raw spectral counts were normalized against total protein counts. A protein fold change (or ratio) between paired samples was calculated by dividing the average spectral counts of triplicate measurements. A ratio (Δ*ftsH* over WT) greater or smaller than 1 indicates that levels of a given protein in Δ*ftsH* samples are higher or lower, respectively, than those in respective WT controls. Two-tailed paired Student’s t test was performed on data from three replicates. Differences with average fold changes ≥1.5 and *p* value ≤ 0.05 were considered significant.

### RNA-seq analysis

Overnight culture in TSB was diluted 100 times in a fresh TSB and incubated in a shaking incubator at 37 °C for 8 h. After immediate stabilization of RNA in all samples by RNAprotect Bacteria Reagent (Qiagen), cells were collected by centrifugation, suspended in TE buffer (10 mM Tris HCl, pH 8.0, 1 mM EDTA) and treated with lysostaphin (50 µg/mL final concentration) at 37 °C for 10 min. From the lysed cells, total RNA was isolated with RNeasy Mini Kit (Qiagen) according to the manufacturer’s recommendations. The isolated RNA was sent to the Center for Genomics and Bioinformatics at Indiana University. Sequencing libraries were constructed using the ScriptSeq Complete Kit for Bacteria (Epicentre). The statistical analysis of the RNA-seq results was done with DeSeq. 2 as described previously^49^. The RNA-seq results were deposited in GEO (Gene Expression Omnibus) with the accession number GSE89791.

### Real-time quantitative reverse transcription-PCR (qRT-PCR)

Cells were grown as described above in 1.5 mL TSB, harvested and broken with a Mini-Beadbeater (Biospec Products) at maximum speed for 30 s. After incubation on ice for 5 min, the samples were centrifuged; and the supernatant was used to isolate total RNA according to the manufacturer’s instructions (Qiagen). After DNase treatment with a TURBO DNA-free TM kit (Ambion), approximately 2 μg of total RNA were reverse-transcribed with a PrimeScript RT reagent kit (Qiagen). The cDNA was used as a template for real-time PCR using SYBR-green PCR reagents (Roche). Reactions were performed in a MicroAmp Optical 96-well reaction plate using a 7500 Sequence Detector (Applied Biosystems). Primers used are listed in Supplementary Table 8. All RT-PCR experiments were performed in triplicate with *gyrB* as an internal control. All experiments were repeated independently at least three times with similar results.

### Cell-aggregation assay

The test strains were grown as described above for the proteomic study. Then the cultures were left standing on a bench at room temperature for 4 h. To quantify cell-aggregation, 100 μl of culture was removed, and OD_600_ was measured in a 96 well plate using a Synergy 2 microtiter plate reader (BioTek). Percent aggregation was calculated according to the formula:

$${\rm{ \% }}\,{\rm{a}}{\rm{g}}{\rm{g}}{\rm{r}}{\rm{e}}{\rm{g}}{\rm{a}}{\rm{t}}{\rm{i}}{\rm{o}}{\rm{n}}=([{{\rm{O}}{\rm{D}}}_{{\rm{t}}{\rm{o}}{\rm{t}}{\rm{a}}{\rm{l}}}-{{\rm{O}}{\rm{D}}}_{{\rm{u}}{\rm{p}}{\rm{p}}{\rm{e}}{\rm{r}}{\rm{p}}{\rm{h}}{\rm{a}}{\rm{s}}{\rm{e}}}]/{{\rm{O}}{\rm{D}}}_{{\rm{t}}{\rm{o}}{\rm{t}}{\rm{a}}{\rm{l}}})\times 100$$

### Western blot analysis

Western blot analysis of proteins was carried out as described previously^50^. The SaeS antibody was generated by our laboratory. The FtsH antibody was generated by GLbiochem, China, while all other antibodies were generated by GeneScript.

### Expression of FtsH substrate proteins in *S*. *aureus*

To express select FtsH substrate proteins in *S*. *aureus*, the genes *saeQ*, *murG*, cydA, *ffh*, *hemA*, and *hrtB* were PCR-amplified with the following primer pairs: P592/593, P570/571, PL72/73, P574/575, P576/577, and PL74/75 (Supplementary Table 8). The amplified fragments were digested with either *Eco*RV/*Kpn*I (for *saeQ*, *murG*, *cydA*, *ffh*, and *hrtB*) or *Nco*I*/Kpn*I (for *hemA*). Then they were inserted into the multi-copy plasmid pYJ335 such that the genes are transcribed by an anhydrotetracyline (ATc)-inducible promoter^51^. The resulting plasmids (i.e., pYJ-SaeQ-his, pYJ-MurG-his, pYJ-CydA-his, pYJ-Ffh-his, pYJ-HemA-his, and pYJ-HrtB-his) were inserted into *E*. *coli* DH5a, and then into *S*. *aureus* RN4220. Finally, the plasmids were transduced by ϕ85 into wild type and *ftsH*-deletion mutant of *S*. *aureus*. The test strains at the exponential growth phase in TSB containing erythromycin (10 μg ml^−1^) were incubated in the presence of ATc (100 ng ml^−1^) until early stationary growth phase as described above for proteome analysis. In Western blot analysis, SaeQ was detected by anti-SaeQ antibody whereas other proteins were detected by anti-His-tag antibody.

### Co-purification assay

Flag-tagged FtsH^H431A^ or Flag-tagged SaeS (a negative control for protein interaction) was co-expressed in NMΔ*ftsH* with His-tagged substrate proteins (MurG, Ffh, and HemA) or a control protein (SrtA). Flag-tagged proteins were constitutively produced from pCL55 while the His-tagged substrate proteins were produced from pYJ335 by ATc-mediated induction. Test strains at the exponential growth phase in TSB were incubated in the presence of ATc (100 ng mL^−1^) until early stationary growth phase as described above for proteome analysis. The cell pellets were collected by centrifugation and treated by lysostaphin (50 μg mL^−1^) in Tris-HCl (pH 8.0) at 37 °C for 30 min. Then the proteins were sonicated in 1 mL lysis buffer (10 mM Tris, pH 7.4, 500 mM NaCl, 5% glycerol, pH 7.4) for 10 s. The supernatant was collected, and the substrate proteins were purified with Ni-NTA agarose (Novagen) according to the manufacturer’s instruction. Purified proteins were analyzed by Western blot analysis with anti-Flag antibody (for FtsH^H431A^ and SaeS) or anti-His-tag (for substrate proteins and SrtA).

### Expression and purification of FtsH proteins

To produce the cytoplasmic domain of FtsH with His_6_-tag at the C-terminus (FtsHc = FtsHΔ1–150), the coding sequence from 151a.a. to 697 a.a was PCR-amplified by Phusion DNA polymerase (NEB) with the primer P484/PL111 (Supplementary Table 8). The PCR product was digested with *BamH*I and *Eco*RI and ligated with pET28a treated with the same enzymes, resulting in pET28a-*ftsHc*. To produce GST-FtsHc, the fusion protein of glutathione S-transferase and the cytoplasmic domain of FtsHc, the *ftsH* sequence was cut from pET28a-*ftsH*c with *BamH*I and *Eco*RI treatment, and ligated with pGEX-4T1 treated with the same enzymes. The resulting plasmids were transformed into *E*. *coli* BL21 (DE3). To express the protein, the BL21(DE3) was grown at 37 °C until OD_600_ = 0.6, supplemented with isopropyl-β-D-1-thiogalactopyranoside (IPTG) to 0.5 mM, and further incubated at 16 °C for 16 h. The expressed FtsHc and GST-FtsHc were purified by Nickel column chromatography (Qiagen) or GST Bind^TM^ Kits (Novagen).

### Preparation of membrane vesicles harboring the FtsH and SaeQ protein

Membrane vesicles were purified from the strains that overexpress either FtsH or SaeQ. To overexpress FtsH, the vector pYJ335 was PCR-amplified with the primers P371/372, while the *ftsH* gene including the promoter sequence was amplified with the primers P39/370 (Supplementary Table 8). The plasmid was assembled with LIC as described above. The plasmid was inserted into *E*. *coli* DH5a, and then into *S*. *aureus* RN4220. Finally, the plasmid was transduced by ϕ85 into wild type of *S*. *aureus* Newman. For SaeQ protein, we used the NMΔ*ftsH* strain with pYJ-SaeQ-his plasmid. The membrane vesicles were prepared from the test strains as described previously^30^. The protein concentration in the membrane vesicle suspension was determined by the bicinchoninic acid assay (Yeasen Bio, China). The membrane vesicles were stored at -80 °C until use.

### Expression and purification of FtsH substrate proteins

To produce the putative FtsH substrate proteins (MurG, Ffh, and HemA) with His_6_-tag at the C-terminus, the corresponding genes were PCR-amplified by Phusion DNA polymerase (NEB) with the following primer sets: P601/602 (*murG*), P603/604 (*ffh*), and P605/606 (*hemA*) (Supplementary Table 8). The PCR products were digested with *BamH*I and *Eco*RI and inserted into pET28a and transformed into *E*. *coli* BL21 (DE3). The resulting BL21(DE3) was grown at 37 °C until OD_600_ = 0.6, supplemented with 5 mM isopropyl-b-D-thiogalactopyranoside (IPTG), and further incubated at 16 °C overnight. The expressed proteins were purified with Ni-NTA agarose (Novagen) according to the manufacturer’s instruction. Proteins were eluted with 500 mM imidazole prepared in lysis buffer (10 mM Tris, pH 7.4, 500 mM NaCl, 5% glycerol, pH 7.4). Purification of MBP-SaeS, MBP-SaeS L18P and His-SaeSc (SaeSΔ1–92) were carried out as previously reported^29,52^.

All purified proteins were dialyzed against the dialysis buffer (10 mM Tris HCl, pH 7.5, 15% (w/v) glycerol, 50 mM KCl, 0.5% (w/v) Nonidet P-40, 5 mM MgCl_2_, 1 mM dithiothreitol).

### Protein degradation assay

#### SaeQ

The membrane vesicles harboring FtsH (300 μg) and SaeQ (100 μg) were incubated in the presence of 5 mM MPOS (pH 7.0), 0.5 M KCl, 12.5% PEG3350 and 250 mM DTT at 37 °C for 10 min^53^. The mixture were diluted 10-fold with degradation buffer (50 mM Tris HCl [pH 8.0], 20 mM KCl, 5 mM MgCl_2_, 12.5 μM Zn(OAc)_2_, 10% (w/v) glycerol) at 42 °C. The reaction was initiated by the addition of 8 mM ATP. At 0, 30, 60, 90, and 120 min, an aliquot (15 μL) was withdrawn, mixed with 400 μL 20% trichloroacetic acid (TCA), and placed at -20 °C for 30 min. After centrifugation, the protein pellet was washed with ice-cold acetone twice, and dissolved in 1× SDS-PAGE sample buffer (30 μL). Samples were heated at 95 °C for 5 min and subjected to 8–15% SDS-PAGE and Western blot analysis with anti-SaeQ antibody.

#### Other purified substrate proteins

The membrane vesicles harboring FtsH (300 μg) were mixed with purified substrates (10 μg) in 100 μL of reaction buffer (50 mM Tris HCl [pH 8.0], 20 mM KCl, 5 mM MgCl_2_, 12.5 μM Zn(OAc)_2_, 10% (w/v) glycerol, 1 mM dithiothreitol) at 42 °C^54^. The reaction was initiated by the addition of 8 mM ATP. At 0, 30, 60, 90, and 120 min post addition of ATP, the reaction was stopped by mixing an aliquot (15 μL) with 2× SDS-PAGE sample buffer (15 μL). Samples were heated at 95 °C for 5 min and subjected to 8%-15% SDS-PAGE and Western blot analysis with anti-His_6_-tag antibody.

### Animal test

Bacterial cells were grown in TSB to exponential growth phase (OD_600_ = 1.0) and then washed with and suspended in PBS to OD_600_ = 1.0. The bacterial suspension (100 µl, ~ 2.5 × 10^7^ cfu) was administered into 8 female 6 week-old Balb/c mice via retro-orbital injection. The infected mice were watched for 7 days. The survival curves were compared by Log-rank (Mantel-Cox) test with Prism 5 (GraphPad). To measure colony forming units of *S*. *aureus* in kidneys, at day 4 post infection, 8 mice are euthanized, and kidneys were harvested, ground, diluted, and spread on TSA. The plates were incubated at 37 °C overnight; then colonies were enumerated. The animal experiment was performed by following the Guide for the Care and Use of Laboratory Animals of the National Institutes of Health. The animal protocol was approved by the ethics committee of Renji Hospital, School of Medicine, Shanghai Jiaotong University (Protocol # RJ-M-2014-0305). Every effort was made to minimize suffering of the animals.

## Electronic supplementary material

Supplementary Information
