# Author Correction: Rewiring of the FtsH regulatory network by a single nucleotide change in *saeS* of *Staphylococcus aureus*

**DOI:** 10.1038/s41598-020-73764-z

**Published:** 2020-10-13

**Authors:** Qian Liu, Mo Hu, Won-Sik Yeo, Lei He, Tianming Li, Yuanjun Zhu, Hongwei Meng, Yanan Wang, Hyunwoo Lee, Xiaoyun Liu, Min Li, Taeok Bae

**Affiliations:** 1 Department of Laboratory Medicine, Ren Ji Hospital, School of Medicine, Shanghai Jiao Tong University, Shanghai, 200127 China; 2 Institute of Analytical Chemistry and Synthetic and Functional Biomolecules Center, College of Chemistry and Molecular Engineering, Peking University, Beijing, 100871 China; 3 Department of Microbiology and Immunology, Indiana University School of Medicine-Northwest, Gary, IN 46408 USA; 4 Department of Biopharmaceutical Sciences and Center for Biomolecular Sciences, College of Pharmacy, University of Illinois at Chicago, Chicago, IL 60607 USA; 5 Present Address: Johnson Medical Company Advanced Energy, Shanghai, 200233 China

Correction to: *Scientific Reports* 10.1038/s41598-017-08774-5, published online 16 August 2017

Due to an annotation error, the gene and protein name for the processive diacylglycerol beta-glucosyltransferase in this Article is listed as *murG*/MurG. The correct names are *ypfP*/YpfP.

Therefore, all instances of “*murG*” and “MurG” in the Article should read “*ypfP*” and “YpfP”, respectively.

