# Supplementary material for: Rewiring of the FtsH regulatory network by a single nucleotide change in saeS of Staphylococcus aureus
Source: Sci Rep. 2017 Aug 16;7:8456. doi: 10.1038/s41598-017-08774-5 (PMC5559551; doi:10.1038/s41598-017-08774-5)
Supplement: Supplementary file 1 — Supplementary Information [file 41598_2017_8774_MOESM1_ESM.pdf]

Supplementary Information

Rewiring of the FtsH regulatory network by a single nucleotide change in *saeS* of *Staphylococcus aureus*

Qian Liu[1#], Mo Hu[2#], Won-Sik Yeo[3], Lei He[1], Tianming Li[1], Yuanjun Zhu[1&], Hongwei Meng[1], Yanan Wang[1], Hyunwoo Lee[4], Xiaoyun Liu[2*], Min Li[1*] and Taeok Bae[3*]

Affiliations:

1. Department of Laboratory Medicine, Ren Ji Hospital, School of Medicine, Shanghai Jiao Tong University, Shanghai 200127, China

2. Institute of Analytical Chemistry and Synthetic and Functional Biomolecules Center, College of Chemistry and Molecular Engineering, Peking University, Beijing 100871, China

3. Department of Microbiology and Immunology, Indiana University School of Medicine-Northwest, Gary, Indiana, 46408, USA

4. Department of Biopharmaceutical Sciences and Center for Biomolecular Sciences, College of Pharmacy, University of Illinois at Chicago, Chicago, Illinois 60607, USA

[&] Current address: Johnson Medical Company Advanced Energy, Shanghai 200233, China

# These authors contributed equally to this work.

[*] Corresponding author

E-mail: xiaoyun.liu@pku.edu.cn (XL), ruth_limin@126.com (ML) and tbae@iun.edu (TB)

Supplementary Table 1. Proteins increased in abundance by *ftsH*-deletion in Newman (Red, Increased in both strain backgrounds)

Gene ID	USA300	Name	WT	*ftsH*	*ftsH*/WT	*p* value	Protein Function
NWMN_0049	107		7.09	13.36	1.9	0.0073	hypothetical protein
NWMN_0196	256	*lrgA*	0.5	1.77	3.54	0.0205	murein hydrolase regulator LrgA
NWMN_0197	257	*lrgB*	0.5	1.78	3.57	0.0062	antiholin-like protein LrgB
NWMN_0222	282		1.77	4.1	3.29	0.0405	hypothetical protein
NWMN_0252	310		4.85	13.4	3.09	0.0101	hypothetical protein
NWMN_0271			3.04	5.82	2.07	0.0437	hypothetical protein
NWMN_0278			0.5	1.78	3.57	0.0062	hypothetical protein
NWMN_0281			3.38	6.17	1.84	0.0031	hypothetical protein
NWMN_0397	404	*set10nm*	0.5	1.45	2.89	0.0144	superantigen-like protein
NWMN_0474	490	*hslO*	13.59	103.8	7.7	0.0003	Hsp33-like chaperonin
NWMN_0529	551		19.03	28.43	1.52	0.026	putative GTP cyclohydrolase
NWMN_0559	579		0.5	1.85	3.71	0.0172	hypothetical protein
NWMN_0585	602		13	23.58	1.89	0.028	hypothetical protein
NWMN_0624	641	*lipA*	0.76	2.54	3.58	0.0097	lipase/esterase LipA
NWMN_0674	690	*saeS*	46.1	156.74	3.43	0.0005	sensor histidine kinase SaeS
NWMN_0676	692	*saeQ*	35.96	58.44	1.64	0.0089	SaeS-regulatory protein SaeQ
NWMN_0687	703		14.02	24.38	1.78	0.0146	hypothetical protein NWMN_0687
NWMN_0691	707		15.4	25.87	1.68	0.0273	ABC transporter permease
NWMN_0758	774	*emp/ssp*	34.18	60.3	1.76	0.0049	extracellular matrix binding protein
NWMN_0816	849	*mnhG*	0.93	2.19	2.36	0.0001	monovalent cation/H+ antiporter
NWMN_0851	883		14.85	25.21	1.83	0.0405	MHC class II analog protein
NWMN_0887	918		0.67	6.95	10.99	0.0012	diacylglycerol glucosyltransferase
NWMN_0917	950	*sspB*	0.5	4.13	8.26	0.0289	cysteine protease precursor
NWMN_0952	986	*cydA*	16.5	28.34	1.72	0.0003	cytochrome D ubiquinol oxidase, subunit I
NWMN_0981	1016	*cyoD**	0.5	5.99	11.98	0.0015	protoheme IX farnesyltransferase
NWMN_0988	1024	*coaD*	4.69	8	1.83	0.0367	phosphopantetheine adenylyltransferase
NWMN_1076	1060		0.63	1.77	2.66	0.0078	superantigen-like protein
NWMN_1118	1100		5.57	9.45	1.64	0.039	hypothetical protein
NWMN_1147	1130	*ffh*	30.39	49.13	1.63	0.0055	signal recognition particle protein
NWMN_1175	1158		0.76	1.78	2.37	0.0002	hypothetical protein
NWMN_1261	1245		7.64	17.47	2.33	0.0072	glycine betaine transporter 1
NWMN_1334	1316	*msrB*	3.08	5.76	1.89	0.0138	methionine sulfoxide reductase B
NWMN_1371	1351		22.74	51.48	2.31	0.0062	hypothetical protein
NWMN_1444			0.5	1.1	2.19	0.0023	hypothetical protein
NWMN_1465	1522	*dnaG*	7.68	12.65	1.64	0.006	DNA primase
NWMN_1500	1557		8.27	12.35	1.51	0.0357	HAD superfamily hydrolase
NWMN_1516	1572		0.63	2.12	3.36	0.0053	hypothetical protein

NWMN_1524	1579		3.72	7.18	1.98	0.0226	aminotransferase, class V
NWMN_1566	1619	*hemA*	0.5	19.11	38.22	0	glutamyl-tRNA reductase
NWMN_1621	1674	*htrA*	31.83	53.4	1.71	0.0192	putative serine protease HtrA
NWMN_1709	1760	*bsaG*	0.93	2.87	3.12	0.0103	lantibiotic ABC transporter protein
NWMN_1727	1785		2.79	5.12	1.84	0.0031	hypothetical protein NWMN_1727
NWMN_1732	1789		2.4	5.77	3.11	0.0332	hypothetical protein
NWMN_1823	1866	*vraS*	3.97	8.36	2.24	0.0291	sensor histidine kinase VraS
NWMN_1843	1886	*pcrA*	24.02	34.78	1.51	0.0363	ATP-dependent DNA helicase
NWMN_1872	1917	*eap/map*	1407.71	2231.69	1.58	0.0005	MHC class II analog protein
NWMN_1873	1918		0.5	2.2	4.41	0.0209	truncated beta-hemolysin, partial
NWMN_1877	1920	*chp*	0.63	2.12	3.64	0.0152	chemotaxis-inhibiting protein CHIPS
NWMN_1938	1983	*groES*	7.34	11.51	1.58	0.0027	co-chaperonin GroES
NWMN_1943	1989	*agrB*	2.78	5.14	1.93	0.0223	accessory gene regulator protein B
NWMN_2109	2164		1.22	5.06	4.34	0.0013	MHC class II analog protein
NWMN_2170	2221	*moaD*	0.92	3.21	3.99	0.0036	molybdopterin synthase small subunit
NWMN_2215	2262		0.63	2.8	4.99	0.0224	hypothetical protein
NWMN_2261	2306	*hrtA*	0.5	17.49	34.98	0	ABC transporter ATP-binding protein
NWMN_2262	2307	*hrtB*	1.52	6.35	5.68	0.0276	ABC transporter permease
NWMN_2529	2565	*clfB*	52.8	79.18	1.5	0.0052	clumping factor B precursor
NWMN_2602	2637		0.63	2.94	4.99	0.0034	hypothetical protein
NWMN_2606	2640		0.5	58.69	117.39	0	putative transcription regulator

* *cyoE* in USA300

Supplementary Table 2. Proteins decreased in abundance by *ftsH*-deletion in Newman (Red, Decreased in both strain backgrounds)

Gene ID	USA300	Name	WT	*ftsH*	*ftsH*/WT	*p* value	Protein Function
NWMN_0094	151	*adhE*	73.82	49.04	0.67	0.0321	acetaldehyde-CoA/alcohol dehydrogenase
NWMN_0097	154	*capC*	4.85	2.81	0.55	0.0275	capsular polysaccharide synthesis enzyme CapC
NWMN_0103	160	*capI*	2.74	1.43	0.55	0.0108	capsular polysaccharide biosynthesis protein CapI
NWMN_0109	166	*capO*	6.07	1.93	0.31	0.0418	capsular polysaccharide biosynthesis protein CapO
NWMN_0134	192		1.81	0.5	0.32	0.017	hypothetical protein
NWMN_0135	193	*murQ*	1.81	0.5	0.32	0.017	N-acetylmuramic acid-6-phosphate etherase
NWMN_0136	194	*ptsG*	1.86	0.67	0.37	0.0255	sucrose-specific PTS transporter
NWMN_0141	198		8.27	5.4	0.65	0.0064	hypothetical protein
NWMN_0149	206		12.71	5.07	0.41	0.0059	azoreductase
NWMN_0158	216	*uhpT*	1.56	0.5	0.36	0.0241	sugar phosphate antiporter
NWMN_0169	227	*fadD*	1.52	0.5	0.35	0.0114	acyl-CoA dehydrogenase FadD-like protein
NWMN_0193	253	*scdA*	6.75	2.88	0.45	0.0184	cell wall biosynthesis protein ScdA
NWMN_0220	279		2.11	0.67	0.35	0.0112	hypothetical protein
NWMN_0289			1.22	0.5	0.45	0.0224	phage terminase large subunit
NWMN_0294			14.86	5.47	0.37	0.0004	phage major head protein
NWMN_0306			7.55	2.86	0.4	0.0304	hypothetical protein
NWMN_0374	382		7.39	4.37	0.59	0.0306	sodium:dicarboxylate symporter family protein
NWMN_0389	394	*set2nm*	1.22	0.68	0.55	0.0046	superantigen-like protein
NWMN_0391	398	*set4nm*	0.93	0.5	0.54	0.0028	superantigen-like protein
NWMN_0439	449	*treC*	6.41	1.43	0.23	0.0027	alpha,alpha-phosphotrehalase
NWMN_0444	454	*recR*	1.81	0.85	0.49	0.0143	recombination protein RecR
NWMN_0452	466		3.92	2.47	0.63	0.0133	hypothetical protein
NWMN_0457	471		3.38	1.78	0.52	0.016	hypothetical protein
NWMN_0473	489	*ftsH*	118.43	1.66	0.01	0	ATP-dependent metalloprotease FtsH
NWMN_0476	492	*folP*	10.72	7.12	0.65	0.0298	dihydropteroate synthase
NWMN_0480	503		2.7	0.5	0.26	0.0239	GntR family regulatory protein
NWMN_0515	538	*capD*	12.37	4.61	0.38	0.0243	polysaccharide biosynthesis protein CapD
NWMN_0535	557		4.01	1.53	0.36	0.0396	hypothetical protein
NWMN_0574	591		3.42	2.19	0.65	0.0239	acetyltransferase, GNAT family protein
NWMN_0584	601		27.48	17.23	0.62	0.0284	hydrolase
NWMN_0601	618		62.79	29.27	0.47	0.003	hypothetical protein
NWMN_0603	620		11.14	6.51	0.59	0.0116	ABC transporter ATP-binding protein
NWMN_0614	631		3.46	1.5	0.45	0.0263	hypothetical protein
NWMN_0619	636		9.2	5.75	0.62	0.0037	dihydroxyacetone kinase subunit DhaK
NWMN_0651	668		30.65	19.47	0.64	0.0409	hypothetical protein
NWMN_0695	711		19.5	11.6	0.6	0.0124	hypothetical protein
NWMN_0729	741	*lgt*	3.67	1.43	0.4	0.0251	prolipoprotein diacylglyceryl transferase
NWMN_0746	761		2.11	1.01	0.45	0.0235	hypothetical protein

NWMN_0777	793		4.6	1.77	0.37	0.0254	hypothetical protein
NWMN_0783	816		12.45	6.99	0.49	0.0198	CsbD-like superfamily protein
NWMN_0885	916		22.92	14.1	0.62	0.0138	hypothetical protein
NWMN_0906	939		30.8	20.44	0.66	0.0096	glycosyl transferase, group 1 family protein
NWMN_0933	966	*purE*	26.08	12.02	0.47	0.0036	phosphoribosylaminoimidazole carboxylase catalytic subunit
NWMN_0948	982		68.5	42.9	0.64	0.0383	hypothetical protein
NWMN_1018			4.56	1.68	0.29	0.0369	hypothetical protein
NWMN_1022			1.22	0.67	0.54	0.0032	hypothetical protein
NWMN_1025			4.39	3	0.66	0.0428	phage major tail protein
NWMN_1060	1046	*sdhC*	1.56	0.5	0.36	0.0245	succinate dehydrogenase cytochrome b558 subunit
NWMN_1061	1047	*sdhA*	51.86	29.35	0.57	0.0048	succinate dehydrogenase flavoprotein subunit
NWMN_1072	1057		4.98	2.19	0.44	0.0016	hypothetical protein
NWMN_1084	1068		2.4	1.54	0.62	0.0397	phenol soluble modulin beta 1
NWMN_1105	1088		1.86	0.74	0.39	0.0291	glyoxalase
NWMN_1109	1091	*pryR*	8.7	5.42	0.64	0.0427	bifunctional pyrimidine regulatory protein PyrR
NWMN_1126	1109	*fmt*	23.63	15.68	0.66	0.0409	methionyl-tRNA formyltransferase
NWMN_1162	1145	*xerC*	1.56	0.5	0.36	0.0245	tyrosine recombinase xerC
NWMN_1242	1228	*thrB*	2.15	0.67	0.37	0.0243	homoserine kinase
NWMN_1275	1258		1.52	0.67	0.45	0.0235	4-oxalocrotonate tautomerase
NWMN_1290	1273		1.22	0.68	0.55	0.0046	oligopeptide ABC transporter ATP-binding protein
NWMN_1325	1305	*sucB*	55.46	36.77	0.67	0.0297	dihydrolipoamide succinyltransferase
NWMN_1327	1307	*arlS*	1.22	0.5	0.45	0.0224	two-component sensor histidine kinase
NWMN_1328	1308	*arlR*	10.42	6.45	0.62	0.0139	two-component response regulator
NWMN_1398	1440		3.76	0.68	0.19	0.0051	hypothetical protein
NWMN_1411	1453		3.67	2.12	0.56	0.0347	hypothetical protein
NWMN_1414	1456	*malA*	17.72	9.4	0.54	0.0101	alpha-D-1,4-glucosidase
NWMN_1415	1457	*marR*	9.88	3.21	0.33	0.0202	maltose operon transcriptional repressor
NWMN_1453	1510		2.49	0.68	0.32	0.0393	5-formyltetrahydrofolate cyclo-ligase
NWMN_1459	1516		7.68	3.22	0.42	0.0068	ABC transporter ATP-binding protein
NWMN_1474	1531	*phoH*	22.91	15.31	0.66	0.0357	phosphate starvation-induced protein,
NWMN_1493	1550		2.15	1.1	0.53	0.0226	hypothetical protein
NWMN_1578	1631	*dnaB*	4.26	1.59	0.36	0.0413	replication initiation protein
NWMN_1579	1632	*nrdR*	10.33	4.75	0.48	0.0423	transcriptional regulator NrdR
NWMN_1580	1633	*gapB*	47.02	19.77	0.42	0.0011	glyceraldehyde 3-phosphate dehydrogenase 2
NWMN_1603	1655	*ald*	15.83	8.34	0.53	0.0226	alanine dehydrogenase
NWMN_1614	1667		1.52	0.68	0.46	0.0275	glycerophosphoryl diester phosphodiesterase
NWMN_1626	1679	*acs*	18.73	8.62	0.45	0.0385	acetyl-CoA synthetase
NWMN_1627	1680	*acuA*	1.22	0.5	0.45	0.0228	acetoin utilization protein
NWMN_1658	1711	*putA*	12.57	4.78	0.41	0.0308	proline dehydrogenase
NWMN_1681	1731	*pckA*	126.4	76.58	0.61	0	phosphoenolpyruvate carboxykinase
NWMN_1689	1740		15.45	4.05	0.26	0.0011	hypothetical protein
NWMN_1699	1751	*hsdS*	4.31	2.11	0.45	0.0348	type I restriction modification system

NWMN_1749	1807		2.4	0.67	0.34	0.0235	glutamine transport ATP-binding protein
NWMN_1812			25.69	14.74	0.57	0	phage repressor
NWMN_1847	1890	*sspB*	3.38	0.5	0.15	0.0001	staphopain thiol proteinase
NWMN_1922	1970		10.72	4.96	0.45	0.0286	phage exonuclease
NWMN_1931	1978		1.22	0.5	0.45	0.0228	ABC transporter iron compound-binding protein
NWMN_1970	2022	*sigB*	10.58	5.67	0.54	0.0482	RNA polymerase sigma factor SigB
NWMN_2016	2066	*upp*	159.83	105.81	0.66	0.0231	uracil phosphoribosyltransferase
NWMN_2039			6.16	2.94	0.48	0.0145	hypothetical protein
NWMN_2060	2108	*mtlD*	68.45	41.6	0.61	0.0032	mannitol-1-phosphate 5-dehydrogenase
NWMN_2061	2109	*fmtB*	3.33	1.78	0.55	0.0071	methicillin resistance determinant FmtB protein
NWMN_2080	2138		16.38	7.05	0.43	0.0176	hypothetical protein NWMN_2080
NWMN_2087	2143		56.38	34.71	0.62	0.0087	hypothetical protein
NWMN_2093	2149	*lacG*	12.28	2.46	0.2	0.0067	6-phospho-beta-galactosidase
NWMN_2096	2152	*lacD*	6.75	0.74	0.12	0.0024	tagatose 1,6-diphosphate aldolase
NWMN_2098	2154	*lacB*	2.79	0.5	0.2	0.003	galactose-6-phosphate isomerase subunit LacB
NWMN_2099	2155	*lacA*	3.42	0.67	0.22	0.011	galactose-6-phosphate isomerase subunit LacA
NWMN_2110	2165	*alsD*	16.29	6.03	0.39	0.0206	alpha-acetolactate decarboxylase
NWMN_2111	2166	*alsS*	43.81	24.02	0.55	0.0021	acetolactate synthase
NWMN_2157	2209		10.85	5.06	0.47	0.0096	GCN5-related N-acetyltransferase
NWMN_2171	2222	*moaE*	2.79	0.91	0.32	0.0099	molybdopterin synthase large subunit
NWMN_2183	2234		4.56	2.12	0.46	0.0025	nucleoside hydrolase family protein
NWMN_2217	2264		13.76	9.25	0.67	0.0344	RpiR family protein
NWMN_2231	2277	*hutI*	3.42	1.1	0.33	0.0026	imidazolonepropionase
NWMN_2232	2278	*hutU*	2.45	0.67	0.27	0.0003	urocanate hydratase
NWMN_2233	2279		3.62	1.2	0.32	0.0035	LysR family regulatory protein
NWMN_2235	2281	*hutG*	27.77	11.96	0.43	0.0034	formimidoylglutamase
NWMN_2245	2290		0.93	0.5	0.54	0.0028	3-methyladenine DNA glycosylase
NWMN_2251	2296		11.85	0.68	0.06	0.0001	esterase-like protein
NWMN_2293	2339		2.2	0.5	0.31	0.0335	hypothetical protein
NWMN_2296	2342	*narH*	4.6	1.63	0.32	0.0236	nitrate reductase beta chain
NWMN_2311	2357		43.01	23.89	0.55	0.0077	amino acid ABC transporter ATP-binding protein
NWMN_2315	2362	*gpmA*	124.9	73.17	0.58	0.0363	phosphoglyceromutase
NWMN_2319	2366	*hlgC*	36.77	23.65	0.64	0.0476	gamma-hemolysin component C
NWMN_2338	2384		2.74	1.43	0.55	0.0108	Na+/H+ antiporter family protein
NWMN_2352	2398		5.66	3.75	0.64	0.024	hypothetical protein
NWMN_2353	2399		14.43	8.69	0.6	0.0203	ABC transporter ATP-binding protein
NWMN_2361	2408	*opp1D*	0.93	0.5	0.54	0.0028	peptide ABC transporter ATP-binding protein
NWMN_2397		*fnbB*	11.06	4.99	0.45	0.0059	fibronectin binding protein B precursor, partial
NWMN_2422	2463		164.47	97.71	0.6	0.0068	D-lactate dehydrogenase
NWMN_2423	2464		1.56	0.5	0.36	0.0245	hydrolase
NWMN_2441	2480		3.38	1.1	0.33	0.003	transcriptional regulator LysR family protein
NWMN_2454	2491		62.17	39.83	0.65	0.0431	1-pyrroline-5-carboxylate dehydrogenase
NWMN_2460	2497		2.11	1.35	0.58	0.0178	aminotransferase, class I

NWMN_2487	2525		24.01	14.7	0.61	0.0426	hypothetical protein
NWMN_2509	2545	*betA*	22.41	11.68	0.53	0.0199	choline dehydrogenase
NWMN_2510	2546	*betB*	37.38	19.79	0.54	0.0348	glycine betaine aldehyde dehydrogenase
NWMN_2523	2558	*nsaS/braS*	0.93	0.5	0.54	0.0028	susceptibility-associated sensor histidine kinase
NWMN_2540	2576		5.4	1.03	0.23	0.0161	PTS system, fructose-specific IIABC component
NWMN_2547	2583		2.74	1.19	0.4	0.0241	glycosyl transferase, group 1 family protein
NWMN_2558	2593		10.43	5.41	0.56	0.0472	hypothetical protein
NWMN_2584	2619		3.63	2.12	0.59	0.0213	hypothetical protein

Supplementary Table 3. Proteins increased in abundance by *ftsH*-deletion in the strain USA300 (Red, Increased in both strain backgrounds)

Gene ID	NM	Name	WT	*ftsH*	*ftsH*/WT	*p* value	Protein Function
SAUSA300_0033		*mecR1*	0.5	14.65	29.3	0	methicillin-resistance MecR1 regulatory protein
SAUSA300_0170	113		91.81	171.52	1.87	0.009	aldehyde dehydrogenase
SAUSA300_0200	143		0.5	1.36	2.72	0.014	peptide ABC transporter ATP-binding protein
SAUSA300_0219	161		0.93	2.37	2.85	0.015	putative iron compound A C transporter
SAUSA300_0237	178		0.67	2.04	3.37	0.024	inosine-uridine preferring nucleoside hydrolase
SAUSA300_0257	197	*lrgB*	0.5	1.39	2.78	0.021	antiholin-like protein LrgB
SAUSA300_0310	252		4.55	15.57	3.56	0.004	hypothetical protein
SAUSA300_0338	330		1.1	4.14	4.26	0.016	glyoxalase family protein
SAUSA300_0350			1.31	2.75	2.32	0.03	Cro/CI family transcriptional regulator-like protein
SAUSA300_0352	343		7.43	22.08	2.99	0.001	ABC transporter ATP-binding protein
SAUSA300_0377	369		14.15	22.81	1.63	0.026	hypothetical protein
SAUSA300_0393	386		0.5	1.75	3.49	0.018	hypothetical protein
SAUSA300_0443	434		0.63	2.43	4.27	0.016	hypothetical protein
SAUSA300_0483	468		10.34	16.16	1.58	0.026	tetrapyrrole methylase family protein
SAUSA300_0490	474	*hslO*	18.15	117.46	6.89	0.001	Hsp33-like chaperonin
SAUSA300_0508	485		2.24	4.47	2.01	0.024	hypothetical protein
SAUSA300_0509	486		12.52	18.62	1.54	0.043	ATP:guanido phosphotransferase
SAUSA300_0526	503		13.11	23.02	1.78	0.009	methyltransferase small subunit
SAUSA300_0579	559		8.97	18.56	2.06	0.008	hypothetical protein
SAUSA300_0592	575		17.89	29.5	1.66	0.005	hypothetical protein
SAUSA300_0603	586		0.63	1.69	2.51	0.009	hypothetical protein
SAUSA300_0692	676	*saeQ*	2.88	13.67	4.8	0	SaeS-regulatory protein SaeQ
SAUSA300_0732	717		4.15	8.58	2.1	0.005	hypothetical protein
SAUSA300_0748	733		15.8	25.51	1.62	0.001	hypothetical protein
SAUSA300_0851	818	*mnhE*	1.1	2.4	2.31	0.025	monovalent cation/H+ antiporter subunit E
SAUSA300_0864	832	*argG*	3.47	9.32	3.25	0.038	argininosuccinate synthase
SAUSA300_0874	842		12.29	20.01	1.63	0.005	hypothetical protein SAUSA300_0874
SAUSA300_0911	880		2.91	6.16	2.14	0.021	monovalent cation:proton antiporter-2
SAUSA300_0918	887		0.8	8.88	11.78	0.001	diacylglycerol glucosyltransferase
SAUSA300_0945	912		2.88	6.57	2.36	0.022	isochorismate synthase family protein
SAUSA300_0958	925		17.29	32.61	1.89	0.002	hypothetical protein
SAUSA300_0964	931		0.5	1.04	2.07	0.001	hypothetical protein
SAUSA300_0986	952	*cydA*	6.16	13.79	2.27	0.009	cytochrome D ubiquinol oxidase, subunit I
SAUSA300_1016	981	*cyoE**	0.5	9.64	19.28	0	protoheme IX farnesyltransferase
SAUSA300_1052	1066		8.07	13.14	1.68	0.031	fibrinogen-binding protein
SAUSA300_1104	1121	*coaBC*	22.71	35.78	1.6	0.018	phosphopantothenoylcysteine decarboxylase /phosphopantothenate-cysteine ligase
SAUSA300_1112	1129		4.82	7.51	1.54	0.029	protein phosphatase 2C domain-containing protein

SAUSA300_1130	1147	*ffh*	31.41	46.84	1.51	0.027	signal recognition particle protein
SAUSA300_1155	1172		7.29	14.29	2	0.007	putative membrane-associated zinc metalloprotease
SAUSA300_1177	1193	*cinA*	6.8	12.48	1.81	0.012	competence/damage-inducible protein cinA
SAUSA300_1191	1207	*glpF*	0.63	1.36	2.15	0	glycerol uptake facilitator
SAUSA300_1228	1242	*thrB*	0.8	1.72	2.14	0	homoserine kinase
SAUSA300_1231	1245		0.97	2.07	2.13	0	gamma-aminobutyrate permease
SAUSA300_1236	1250		1.87	3.05	1.86	0.037	hypothetical protein
SAUSA300_1253	1270	*glcT*	6.05	9.26	1.57	0.035	transcription antiterminator
SAUSA300_1254	1271		5.19	9.97	1.97	0.035	hypothetical protein
SAUSA300_1255	1272	*mprF*	4.82	8.64	1.89	0.033	phosphatidylglycerol lysyltransferase
SAUSA300_1274	1291		0.5	1.04	2.07	0.001	peptide ABC transporter ATP-binding protein
SAUSA300_1293	1311	*lysA*	20.21	30.98	1.55	0.011	diaminopimelate decarboxylase
SAUSA300_1296	1314		0.97	3.05	3.22	0.023	hypothetical protein
SAUSA300_1301	1321		5.38	9.59	1.94	0.031	hypothetical protein
SAUSA300_1344	1364	*dnaD*	4.41	7.87	1.98	0.044	putative DNA replication protein DnaD
SAUSA300_1348	1368		0.97	2.75	2.85	0.007	tRNA CCA-pyrophosphorylase
SAUSA300_1349	1369		3.51	10.04	3.56	0.042	glycosyl transferase, group 1 family protein
SAUSA300_1351	1371		30.21	52.56	1.75	0.002	hypothetical protein
SAUSA300_1355	1375	*aroA*	16.43	24.86	1.55	0.047	3-phosphoshikimate 1-carboxyvinyltransferase
SAUSA300_1447	1405	*xerD*	6.09	9.97	1.66	0.012	tyrosine recombinase XerD
SAUSA300_1449	1407		3.25	4.85	1.51	0.027	MutT/nudix family protein
SAUSA300_1619	1566	*hemA*	0.5	28.2	56.4	0	glutamyl-tRNA reductase
SAUSA300_1632	1579	*nrdR*	7.62	11.61	1.54	0.003	transcriptional regulator NrdR
SAUSA300_1674	1621	*htrA*	49.42	88.96	1.85	0.012	putative serine protease HtrA
SAUSA300_1677	1624		0.5	1.04	2.07	0.001	cell wall surface anchor family protein
SAUSA300_1701	1648		1.1	3.07	2.84	0.015	hypothetical protein
SAUSA300_1851	1762		1.23	2.01	1.9	0.025	hypothetical protein
SAUSA300_1873	1830		21.22	32.8	1.55	0.022	Mur ligase family protein
SAUSA300_1884	1841		21.44	32.52	1.51	0.002	CamS sex pheromone cAM373
SAUSA300_1913	1869		3.47	6.84	2.19	0.033	ABC transporter ATP-binding protein
SAUSA300_1986	1941		3.88	8.58	2.25	0.007	hypothetical protein
SAUSA300_1987	1942		5.08	8.91	1.88	0.029	carbon-nitrogen family hydrolase
SAUSA300_2029	1977		0.63	2.04	3.22	0.011	hypothetical protein
SAUSA300_2046	1994	*oxaA*	5.08	8.94	1.86	0.037	membrane protein insertase oxaA
SAUSA300_2078	2028	*murA*	37.91	58.68	1.55	0.002	UDP-N-acetylglucosamine 1-carboxyvinyltransferase
SAUSA300_2086	2036		4.48	6.89	1.57	0.041	hypothetical protein
SAUSA300_2159	2104		9.26	17.55	1.94	0.016	aldo/keto reductase family protein
SAUSA300_2206	2154		0.5	2.04	4.08	0.016	hypothetical protein
SAUSA300_2285	2239	*galM*	3.21	5.83	1.81	0.001	aldose 1-epimerase
SAUSA300_2306	2261	*hrtA*	0.5	27.65	55.3	0	ABC transporter ATP-binding protein
SAUSA300_2307	2262	*hrtB*	1.17	21.25	26.8	0.001	ABC transporter permease
SAUSA300_2319	2274		1.1	3.41	3.03	0.011	pyridine nucleotide-disulfide oxidoreductase

SAUSA300_2326	2281		5.79	8.61	1.5	0.015	transcription regulatory protein
SAUSA300_2365	2318	*hlgA*	2.24	6.45	2.79	0.008	gamma-hemolysin component A
SAUSA300_2367	2320	*hlgB*	0.5	1.69	3.37	0.024	gamma-hemolysin component B
SAUSA300_2390	2344	*opuCd*	0.63	3.49	5.52	0.021	glycine betaine/carnitine/choline permease
SAUSA300_2391	2345	*opuCc*	12.18	22.91	1.87	0.003	glycine betaine/carnitine/choline ABC transporter
SAUSA300_2393	2347	*opuCa*	26.41	60.88	2.32	0.004	glycine betaine/carnitine/choline ABC transporter ATP-binding protein
SAUSA300_2400	2354		13.59	21.04	1.56	0.008	glutamyl-aminopeptidase
SAUSA300_2441	2399	*fnbA*	0.5	1.04	2.07	0.001	fibronectin binding protein A
SAUSA300_2459	2418		3.85	6.51	1.7	0.002	MarR family transcriptional regulator
SAUSA300_2494	2457		9.27	16.43	1.78	0	copper-translocating P-type ATPase
SAUSA300_2558	2523	*nsaS/braS*	0.8	2.43	3.2	0.01	histidine kinase
SAUSA300_2565	2529	*clfB*	16.4	28.61	1.76	0.011	clumping factor B
SAUSA300_2579	2543		16.73	40.87	2.44	0	N-acetylmuramoyl-L-alanine amidase
SAUSA300_2603	2569	*lip*	24.35	56.97	2.36	0.001	triacylglycerol lipase
SAUSA300_2614	2579		0.5	1.36	2.72	0.014	hypothetical protein
SAUSA300_2633	2598	*vraD*	0.63	1.69	2.51	0.009	bacitracin ABC transporter ATP-binding protein
SAUSA300_2637	2602		0.5	2.66	5.33	0.036	hypothetical protein
SAUSA300_2640	2606		0.67	76.46	130.65	0	putative transcriptional regulator
SAUSA300_2643	2609		11.02	19.62	1.79	0.009	chromosome partioning protein ParB family

* *cyoD* in the strain Newman

Supplementary Table 4. Proteins decreased in abundance by *ftsH* deletion in USA300 (Red, Decreased in both strain backgrounds)

Gene ID	NM	Name	WT	*ftsH*	*ftsH*/WT	*p* value	Protein Function
SAUSA300_0008	7	*hutH*	6.39	1.72	0.28	0.01	histidine ammonia-lyase
SAUSA300_0013	12		0.97	0.5	0.52	0.002	hypothetical protein
SAUSA300_0017	16	*purA*	28.17	16.04	0.58	0.044	adenylosuccinate synthetase
SAUSA300_0027			2.24	0.66	0.29	0.003	hypothetical protein
SAUSA300_0108	50		14.9	7.22	0.49	0.011	myosin-cross-reactive antigen
SAUSA300_0173	115		82.72	52.16	0.63	0.031	hypothetical protein
SAUSA300_0179	121		4.18	1.75	0.42	0.031	formate dehydrogenase
SAUSA300_0194	136		11.21	6.51	0.59	0.024	sucrose-specific PTS transporter protein
SAUSA300_0195	137		4.11	1.64	0.32	0.046	transcriptional regulator
SAUSA300_0263	203	*rbsD*	2.24	1.17	0.53	0.048	D-ribose pyranase
SAUSA300_0435	426		16.17	7.25	0.45	0.001	ABC transporter ATP-binding protein
SAUSA300_0489	473	*ftsH*	136.67	4.14	0.03	0	putative cell division protein FtsH
SAUSA300_0665	648		2.8	0.5	0.28	0.025	acetyltransferase
SAUSA300_0772	756	*clfA*	149.53	98.88	0.67	0.011	clumping factor A
SAUSA300_0916	885		43.58	28.79	0.66	0.036	hypothetical protein
SAUSA300_0966	933	*purE*	37.8	19.03	0.5	0.004	phosphoribosylaminoimidazole carboxylase
SAUSA300_0968	935	*purC*	58.15	34.45	0.59	0.002	phosphoribosylaminoimidazole-succinocarboxamide synthase
SAUSA300_0969		*purS*	12.89	7.93	0.62	0.013	phosphoribosylformylglycinamidine synthase
SAUSA300_0971	937	*purL*	103.46	65.94	0.64	0.006	phosphoribosylformylglycinamidine synthase II
SAUSA300_0975	941	*purH*	57.45	25.21	0.44	0.003	phosphoribosylaminoimidazolecarboxamide formyltransferase/IMP cyclohydrolase
SAUSA300_0976	942	*purD*	45.42	28.21	0.62	0.004	phosphoribosylamine--glycine ligase
SAUSA300_0982	948		68.81	39.58	0.57	0.003	hypothetical protein
SAUSA300_1058	1073	*hla*	9.94	3.14	0.33	0.016	alpha-hemolysin
SAUSA300_1139	1156	*sucD*	153.31	99.97	0.66	0.027	succinyl-CoA synthetase subunit alpha
SAUSA300_1145	1162	*xerC*	4.26	2.43	0.59	0.025	tyrosine recombinase xerC
SAUSA300_1381		*lukF-PV*	2.84	0.5	0.2	0.003	Panton-Valentine leukocidin, LukF-PV
SAUSA300_1516	1459		10.94	7.22	0.66	0.001	ABC transporter ATP-binding protein
SAUSA300_1638	1585	*phoR*	6.72	2.15	0.29	0.047	sensory box histidine kinase PhoR
SAUSA300_1655	1603	*ald*	27.04	15.57	0.58	0.015	alanine dehydrogenase
SAUSA300_1687	1634		8.96	4.44	0.49	0.05	FtsK/SpoIIIE family protein
SAUSA300_1693	1640		2.54	1.36	0.53	0	hypothetical protein
SAUSA300_1731	1681	*pckA*	219.97	137.26	0.62	0.003	phosphoenolpyruvate carboxykinase
SAUSA300_1761	1710	*epiE*	7.36	3.02	0.38	0.049	lantibiotic epidermin immunity protein F
SAUSA300_1762	1711	*epiF*	52.92	29.57	0.56	0.004	lantibiotic epidermin immunity protein F
SAUSA300_1797	1739		16.51	10.75	0.64	0.027	hypothetical protein
SAUSA300_1882	1839	*gatC*	2.31	0.69	0.36	0.03	aspartyl/glutamyl-tRNA amidotransferase subunit C

SAUSA300_1890	1847	*sspB*	12.7	7.48	0.59	0.045	staphopain thiol proteinase
SAUSA300_1938	1896		1.61	0.5	0.34	0.014	phi77 ORF006-like protein capsid protein
SAUSA300_1970	1922		27.49	17.93	0.65	0.002	putative exonuclease
SAUSA300_2128	2070		0.97	0.5	0.52	0.002	putative drug transporter
SAUSA300_2149	2093	*lacG*	22.15	7.16	0.32	0.005	6-phospho-beta-galactosidase
SAUSA300_2150	2094	*lacE*	3.51	1.36	0.41	0.017	PTS system, lactose-specific IIBC component
SAUSA300_2152	2096	*lacD*	9.41	3.4	0.38	0.036	tagatose 1,6-diphosphate aldolase
SAUSA300_2225	2174	*moaC*	9.56	6.16	0.65	0.046	molybdenum cofactor biosynthesis protein MoaC
SAUSA300_2269	2223		1.9	0.5	0.31	0.018	hypothetical protein
SAUSA300_2276	2230		3.21	2.07	0.65	0.023	M20/M25/M40 family peptidase
SAUSA300_2278	2232	*hutU*	33.84	21.25	0.63	0.023	urocanate hydratase
SAUSA300_2294	2249		2.84	1.17	0.4	0.019	hypothetical protein
SAUSA300_2331	2286		19.61	11.13	0.57	0.003	MarR family transcriptional regulator
SAUSA300_2415	2368		5.9	0.88	0.16	0.001	hypothetical protein
SAUSA300_2469	2429	*sdaAA*	5.04	0.85	0.18	0.003	L-serine dehydratase
SAUSA300_2486	2448	*clpL*	436.7	271.12	0.62	0.003	putative ATP-dependent Clp proteinase
SAUSA300_2498	2461	*crtN*	31.94	20.3	0.64	0.044	squalene synthase
SAUSA300_2545	2509	*betA*	40.53	16	0.39	0.017	choline dehydrogenase
SAUSA300_2546	2510	*betB*	60.77	34.29	0.57	0.036	glycine betaine aldehyde dehydrogenase
SAUSA300_2641	2607		1.27	0.66	0.51	0.001	hypothetical protein

Supplementary Table 5. Genes up-regulated or down-regulated in USA300Δ*ftsH*

Gene ID	Name	Fold Change (*ftsH*/WT)	*p* value	Gene Product
**Upregulated**				
SAUSA300_2640		365.9	0.00000	putative transcriptional regulator
SAUSA300_2307	*hrtB*	211.0	0.00000	ABC transporter permease protein
SAUSA300_2306	*hrtA*	127.0	0.00000	ABC transporter ATP-binding protein
SAUSA300_2637		14.2	0.00000	conserved hypothetical protein
SAUSA300_0490	*hslO*	7.2	0.00000	Heat shock protein 33-like protein
SAUSA300_1092	*pyrP*	2.9	0.00042	uracil permease
SAUSA300_0883		2.7	0.00000	putative surface protein
SAUSA300_1093	*pyrB*	2.4	0.00003	aspartate carbamoyltransferase
SAUSA300_1091	*pyrR*	2.4	0.00000	PyrR bifunctional protein
SAUSA300_1095	*carA*	2.3	0.00140	carbamoyl-phosphate synthase small subunit
SAUSA300_1094	*pyrC*	2.1	0.00182	dihydroorotase
SAUSA300_2270	*glvC*	2.1	0.00000	PTS system arbutin-like IIBC component
SAUSA300_2510		2.1	0.00026	conserved hypothetical protein
SAUSA300_0170		2.1	0.00000	aldehyde dehydrogenase
SAUSA300_0380	*ahpC*	2.0	0.00000	Alkyl hydroperoxide reductase subunit C
SAUSA300_0159	*cap5H*	2.0	0.03210	capsular polysaccharide biosynthesis protein Cap5H
SAUSA300_0604		2.0	0.00030	hydrolase alpha/beta hydrolase fold family
SAUSA300_2459		1.9	0.00725	transcriptional regulator MarR family
SAUSA300_2547		1.9	0.00077	conserved hypothetical protein
SAUSA300_2462	*frp*	1.9	0.00007	NAD(P)H-flavin oxidoreductase
SAUSA300_2089	*pdp*	1.9	0.00001	pyrimidine nucleoside phosphorylase
SAUSA300_2511		1.8	0.00014	conserved hypothetical protein
SAUSA300_1728		1.8	0.00000	oxidoreductase aldo/keto reductase family
SAUSA300_2474		1.8	0.00005	conserved hypothetical protein
SAUSA300_1330	*ilvA*	1.8	0.02787	threonine dehydratase
SAUSA300_0379	*ahpF*	1.8	0.00000	alkyl hydroperoxide reductase subunit F
SAUSA300_2473		1.8	0.00004	conserved hypothetical protein
SAUSA300_2494		1.8	0.00005	copper-translocating P-type ATPase
SAUSA300_1601	*rpmA*	1.8	0.00070	50S ribosomal protein L27
SAUSA300_1163	*rbfA*	1.8	0.00484	ribosome-binding factor A
SAUSA300_2104	*glmS*	1.7	0.00015	glucosamine-fructose-6-phosphate aminotransferase
SAUSA300_0747	*trxB*	1.7	0.00041	thioredoxin-disulfide reductase
SAUSA300_2024	*rsbV*	1.7	0.00030	anti-sigma-B factor antagonist
SAUSA300_2626		1.7	0.00077	conserved hypothetical protein

SAUSA300_0157	*cap5F*	1.7	0.03551	capsular polysaccharide biosynthesis protein Cap5F
SAUSA300_2495		1.7	0.00077	copper chaperone copZ
SAUSA300_2394		1.7	0.00037	conserved hypothetical protein
SAUSA300_0637		1.7	0.00077	dihydroxyacetone kinase DhaL subunit
SAUSA300_1215		1.7	0.01823	conserved hypothetical protein
SAUSA300_2588		1.7	0.03053	preprotein translocase SecY protein
SAUSA300_0566		1.7	0.00052	amino acid permease
SAUSA300_0770		1.7	0.00026	conserved hypothetical protein
SAUSA300_2332		1.7	0.01188	heat shock protein
SAUSA300_0135		1.7	0.00040	Superoxide dismutase (Mn/Fe family)
SAUSA300_2090	*deoC*	1.7	0.00156	deoxyribose-phosphate aldolase
SAUSA300_2304		1.7	0.01188	putative membrane protein
SAUSA300_1342		1.6	0.02887	conserved hypothetical protein
SAUSA300_2529		1.6	0.02262	conserved hypothetical protein
SAUSA300_0636		1.6	0.00086	dihydroxyacetone kinase DhaK subunit
SAUSA300_1698		1.6	0.00155	conserved hypothetical protein
SAUSA300_0355		1.6	0.00169	acetyl-CoA acetyltransferase
SAUSA300_2535	*panE*	1.6	0.00521	2-dehydropantoate 2-reductase
SAUSA300_0695		1.6	0.00115	radical activating enzyme family protein
SAUSA300_0786		1.6	0.00762	OsmC/Ohr family protein
SAUSA300_0161	*cap5J*	1.6	0.01825	capsular polysaccharide biosynthesis protein Cap5J
SAUSA300_1876		1.6	0.00104	DNA polymerase IV
SAUSA300_2485		1.6	0.00558	methylated DNA-protein cysteine methyltransferase
SAUSA300_0771		1.6	0.00188	acetyltransferase GNAT family
SAUSA300_0638		1.6	0.01256	dihydroxyacetone kinase phosphotransfer subunit
SAUSA300_0381		1.6	0.00223	putative NAD(P)H-flavin oxidoreductase
SAUSA300_2550	*nrdG*	1.6	0.04182	anaerobic ribonucleotide reductase small subunit
SAUSA300_2239	*ureB*	1.6	0.00070	urease beta subunit
SAUSA300_2097		1.6	0.00193	conserved hypothetical protein
SAUSA300_1708	*rot*	1.6	0.00885	staphylococcal accessory regulator Rot
SAUSA300_0230		1.6	0.01201	putative membrane protein
SAUSA300_1655	*ald*	1.6	0.00482	alanine dehydrogenase
SAUSA300_2564	*estA*	1.6	0.00276	tributyrin esterase
SAUSA300_2035	*kdpD*	1.6	0.00122	sensor histidine kinase KdpD
SAUSA300_0065	*arcA*	1.6	0.00037	arginine deiminase
SAUSA300_1096	*carB*	1.6	0.01565	carbamoyl-phosphate synthase large subunit
SAUSA300_2404		1.6	0.00232	conserved hypothetical protein
SAUSA300_2625		1.6	0.02486	transcriptional regulator PadR family
SAUSA300_2599	*tetR*	1.6	0.00471	transcription regulator TetR family
SAUSA300_0726		1.6	0.00341	glycerate kinase family protein
SAUSA300_2395		1.5	0.00286	amino acid permease

SAUSA300_1343	*nth*	1.5	0.01286	endonuclease III
SAUSA300_0062	*arcB*	1.5	0.00367	ornithine carbamoyltransferase
SAUSA300_1914		1.5	0.02180	GntR family regulatory protein
SAUSA300_1795		1.5	0.00750	conserved hypothetical protein
SAUSA300_0930		1.5	0.00114	lipoate-protein ligase A family protein
SAUSA300_2238	*ureA*	1.5	0.04927	urease gamma subunit
SAUSA300_0351		1.5	0.02745	putative membrane protein
SAUSA300_0046		1.5	0.02486	conserved hypothetical protein
SAUSA300_0064	*arcD*	1.5	0.01571	arginine/oirnithine antiporter
SAUSA300_2475		1.5	0.01581	conserved hypothetical protein
SAUSA300_2565	*clfB*	1.5	0.00057	clumping factor B
SAUSA300_2244	*ureD*	1.5	0.01569	urease accessory protein UreD
SAUSA300_1910		1.5	0.00638	putative membrane protein
SAUSA300_0234		1.5	0.00706	putative flavohemoprotein
SAUSA300_2367	*hlgB*	1.5	0.00692	gamma-hemolysin component B
SAUSA300_1659	*tpx*	1.5	0.01976	thiol peroxidase
SAUSA300_2251		1.5	0.00706	dehydrogenase family protein
SAUSA300_1296		1.5	0.02842	conserved hypothetical protein
SAUSA300_2240	*ureC*	1.5	0.00794	urease alpha subunit
SAUSA300_1344		1.5	0.02091	putative DNA replication protein DnaD
SAUSA300_2631		1.5	0.00925	putative N-acetyltransferase
SAUSA300_2325		1.5	0.04273	conserved hypothetical protein
SAUSA300_2241	*ureE*	1.5	0.01423	urease accessory protein UreE
SAUSA300_0111		1.5	0.01328	conserved hypothetical protein
SAUSA300_1320	*thyA*	1.5	0.00804	thymidylate synthase
SAUSA300_2219	*moaA*	1.5	0.01102	molybdenum cofactor biosynthesis protein A
SAUSA300_2589		1.5	0.00569	cell wall surface anchor family protein
SAUSA300_0079		1.5	0.00624	putative lipoprotein
SAUSA300_2624		1.5	0.03529	putative membrane protein
SAUSA300_0061	*arcC*	1.5	0.01150	carbamate kinase
SAUSA300_2259		1.5	0.00750	putative transcriptional regulator
SAUSA300_1979		1.5	0.04190	cation transport family protein
SAUSA300_2389		1.5	0.03258	putative drug transporter
SAUSA300_0236		1.5	0.04927	PTS system IIBC components
SAUSA300_2551	*nrdD*	1.5	0.01976	anaerobic ribonucleotide reductase large subunit
SAUSA300_0664		1.5	0.01248	conserved hypothetical protein
SAUSA300_0554		1.5	0.03996	glucosamine-6-phosphate isomerase
SAUSA300_0860	*rocD*	1.5	0.01676	ornithine aminotransferase
SAUSA300_0214		1.5	0.02887	conserved hypothetical protein
SAUSA300_1911		1.5	0.01423	ABC transporter ATP-binding protein
SAUSA300_1912		1.5	0.03168	putative membrane protein

SAUSA300_2226	*moaB*	1.5	0.02786	molybdenum cofactor biosynthesis protein B
SAUSA300_2643		1.5	0.02096	putative chromosome partioning protein ParB family
SAUSA300_2243	*ureG*	1.5	0.00925	urease accessory protein UreG
SAUSA300_2527		1.5	0.03049	conserved hypothetical protein
SAUSA300_2390	*opuCd*	1.5	0.02498	glycine betaine/carnitine/choline permease
SAUSA300_2023	*rsbW*	1.5	0.01987	anti-sigma-B factor serine-protein kinase
SAUSA300_1548		1.5	0.03865	ComE operon protein 2
SAUSA300_0329		1.5	0.02839	putative oxidoreductase
SAUSA300_2478	*cidB*	1.4	0.02022	Holin-like protein cidB
SAUSA300_1216		1.4	0.03837	cardiolipin synthetase
SAUSA300_0078	*copA*	1.4	0.01397	ATPase copper transport
SAUSA300_2593		1.4	0.02096	conserved hypothetical protein
SAUSA300_0394		1.4	0.00762	FAD/NAD(P)-binding Rossmann fold Superfamily
SAUSA300_0308		1.4	0.02096	ABC transporter permease protein
SAUSA300_0352		1.4	0.03027	ABC transporter ATP-binding protein
SAUSA300_0982		1.4	0.03258	conserved hypothetical protein
SAUSA300_2224	*moeA*	1.4	0.00550	molybdopterin biosynthesis protein A
SAUSA300_0288		1.4	0.00188	conserved hypothetical protein
SAUSA300_0041		1.4	0.00750	conserved hypothetical protein
SAUSA300_1913		1.4	0.04844	ABC transporter ATP-binding protein
SAUSA300_1844		1.4	0.04473	bacterioferritin comigratory protein
SAUSA300_2646	*trmE*	1.4	0.01197	tRNA modification GTPase
SAUSA300_2477	*cidC*	1.4	0.04288	pyruvate oxidase
SAUSA300_1843		1.4	0.03902	2-hydroxyacid dehydrogenase family protein
SAUSA300_0106		1.4	0.03210	putative drug transporter
SAUSA300_2227	*moeB*	1.4	0.03850	molybdopterin biosynthesis protein B
SAUSA300_1045	*uvrC*	1.4	0.03209	excinuclease ABC C subunit
SAUSA300_0859		1.4	0.03184	NADH-dependent flavin oxidoreductase
SAUSA300_2145		1.4	0.04927	glycine betaine transporter
SAUSA300_2147		1.4	0.04718	alcohol dehydrogenase zinc-containing
SAUSA300_2603	*lip*	1.4	0.04675	triacylglycerol lipase precursor
SAUSA300_1628	*lysP*	1.4	0.03680	lysine-specific permease
SAUSA300_0781		1.3	0.04723	conserved hypothetical protein
SAUSA300_0042		1.3	0.03018	conserved hypothetical protein
**Down-regulated**				
SAUSA300_0769		0.7	0.04318	putative lipoprotein
SAUSA300_1147	*hslU*	0.7	0.04834	heat shock protein HslVU ATPase subunit HslU
SAUSA300_1011		0.7	0.03084	conserved hypothetical protein
SAUSA300_1076	*mraY*	0.7	0.04314	phospho-N-acetylmuramoyl-pentapeptide-transferase

SAUSA300_2131		0.7	0.04897	conserved hypothetical protein
SAUSA300_2126		0.7	0.02911	drug resistance transporter EmrB/QacA subfamily
SAUSA300_1752	*hsdM*	0.7	0.00915	type I restriction-modification system M subunit
SAUSA300_1584		0.7	0.02059	ATPase AAA family
SAUSA300_0976	*purD*	0.7	0.03996	phosphoribosylamine--glycine ligase
SAUSA300_0277		0.7	0.00885	putative staphyloxanthin biosynthesis protein
SAUSA300_1561		0.7	0.01719	putative membrane protein
SAUSA300_0631		0.7	0.01423	putative nucleoside transporter
SAUSA300_0796		0.7	0.00926	ABC transporter ATP-binding protein
SAUSA300_2136		0.7	0.01328	ABC transporter iron compound-binding protein
SAUSA300_0857		0.7	0.02839	conserved hypothetical protein
SAUSA300_1740		0.7	0.02486	conserved hypothetical protein
SAUSA300_1544	*lepA*	0.7	0.00168	GTP-binding protein LepA
SAUSA300_1985	*sdrH*	0.7	0.00522	serine-aspartate repeat family protein SdrH
SAUSA300_1669		0.7	0.01066	aminotransferase class V
SAUSA300_1440		0.7	0.01987	conserved hypothetical protein
SAUSA300_1106		0.7	0.01125	putative lipoprotein
SAUSA300_2225	*moaC*	0.7	0.00971	molybdenum cofactor biosynthesis protein C
SAUSA300_0801	*seq*	0.7	0.00813	staphylococcal enterotoxin Q
SAUSA300_1074	*ftsL*	0.7	0.00876	cell division protein
SAUSA300_0800	*sek*	0.7	0.00787	staphylococcal enterotoxin K
SAUSA300_2080		0.7	0.04718	conserved hypothetical protein
SAUSA300_1562		0.7	0.00482	LamB/YcsF family protein
SAUSA300_2276		0.7	0.00743	peptidase M20/M25/M40 family
SAUSA300_1965		0.7	0.01642	conserved hypothetical phage protein
SAUSA300_1969		0.6	0.00471	phage transcriptional repressor
SAUSA300_1670	*serA*	0.6	0.00265	D-3-phosphoglycerate dehydrogenase
SAUSA300_0971	*purL*	0.6	0.02477	phosphoribosylformylglycinamidine synthase II
SAUSA300_1201	*glnA*	0.6	0.00529	glutamine synthetase type I
SAUSA300_0733		0.6	0.00123	degV family protein
SAUSA300_0802		0.6	0.01066	conserved hypothetical protein
SAUSA300_1229		0.6	0.00567	hydrolase haloacid dehalogenase-like family
SAUSA300_1372	*recQ*	0.6	0.00776	conserved hypothetical protein
SAUSA300_2092	*dps*	0.6	0.00006	general stress protein 20U
SAUSA300_1739		0.6	0.00795	conserved hypothetical protein
SAUSA300_1072	*mraZ*	0.6	0.00123	protein mraZ
SAUSA300_1200	*glnR*	0.6	0.00471	glutamine synthetase repressor
SAUSA300_0405	*hsdM*	0.6	0.00059	type I restriction-modification system M subunit
SAUSA300_2331		0.6	0.00074	transcriptional regulator MarR family
SAUSA300_2538		0.6	0.00750	amino acid permease family protein
SAUSA300_1564	*accB*	0.6	0.03424	acetyl-CoA carboxylase biotin carboxyl carrier protein

SAUSA300_0798		0.6	0.00133	ABC transporter substrate-binding protein
SAUSA300_0433	*cysM*	0.6	0.00804	cysteine synthase/cystathionine beta-synthase
SAUSA300_2506	*isaA*	0.6	0.03084	immunodominant staphylococcal antigen A precursor
SAUSA300_1496		0.6	0.00071	glycine dehydrogenase subunit 2
SAUSA300_0797		0.6	0.00744	ABC transporter permease protein
SAUSA300_1497		0.6	0.00257	glycine dehydrogenase subunit 1
SAUSA300_1073	*mraW*	0.6	0.00070	S-adenosyl-methyltransferase MraW
SAUSA300_0406		0.6	0.00097	putative restriction specificity protein
SAUSA300_1291		0.6	0.02748	hippurate hydrolase
SAUSA300_1998		0.6	0.00732	putative membrane protein
SAUSA300_2324		0.6	0.01476	PTS system sucrose-specific IIBC component
SAUSA300_1292	*alr2*	0.6	0.00732	alanine racemase
SAUSA300_0898	*spxA*	0.6	0.00089	Regulatory protein spx
SAUSA300_0356		0.6	0.00046	conserved hypothetical protein
SAUSA300_1566		0.6	0.00057	conserved hypothetical protein
SAUSA300_1498	*gcvT*	0.6	0.00012	aminomethyltransferase
SAUSA300_1436		0.6	0.00089	phiSLT ORF144-like protein putative lipoprotein
SAUSA300_0538		0.6	0.00066	NAD dependent epimerase/dehydratase family
SAUSA300_0169		0.6	0.01337	conserved hypothetical protein
SAUSA300_0967	*purK*	0.6	0.00926	phosphoribosylaminoimidazole carboxylase ATPase subunit
SAUSA300_0173		0.6	0.00042	conserved hypothetical protein
SAUSA300_0815	*ear*	0.6	0.01117	Ear protein
SAUSA300_0846		0.6	0.01397	Na+/H+ antiporter family protein
SAUSA300_0445	*gltB*	0.6	0.00624	glutamate synthase large subunit
SAUSA300_1237	*lexA*	0.6	0.00066	LexA repressor
SAUSA300_1563	*accC*	0.6	0.00000	acetyl-CoA carboxylase biotin carboxylase
SAUSA300_0810		0.6	0.00408	conserved hypothetical protein
SAUSA300_0409		0.5	0.00016	conserved hypothetical protein
SAUSA300_0887	*oppB*	0.5	0.00842	oligopeptide ABC transporter permease protein
SAUSA300_1434		0.5	0.00017	phiSLT ORF104a-like protein repressor
SAUSA300_1178	*recA*	0.5	0.00070	recombinase A protein
SAUSA300_2280	*fosB*	0.5	0.00160	metallothiol transferase fosB
SAUSA300_1075	*pbpA*	0.5	0.00000	penicillin-binding protein 1
SAUSA300_0256		0.5	0.00040	holin-like protein lrgA
SAUSA300_0969	*purS*	0.5	0.04273	phosphoribosylformylglycinamidine synthase
SAUSA300_2164		0.5	0.00000	conserved hypothetical protein
SAUSA300_1565		0.5	0.00001	putative urea amidolyase
SAUSA300_1286		0.5	0.00000	aspartate kinase
SAUSA300_1435		0.5	0.00000	phiSLT ORF153-like protein
SAUSA300_1052		0.5	0.00009	fibrinogen-binding protein

SAUSA300_0437		0.5	0.00000	NLPA lipoprotein
SAUSA300_0809		0.5	0.00001	putative DNA primase
SAUSA300_0257	*lrgB*	0.5	0.00000	Antiholin-like protein lrgB
SAUSA300_1014	*pyc*	0.5	0.00000	pyruvate carboxylase
SAUSA300_0360		0.5	0.00053	Cys/Met metabolism PLP-dependent enzyme
SAUSA300_1227	*thrC*	0.5	0.00000	threonine synthase
SAUSA300_0177		0.5	0.00000	conserved hypothetical protein
SAUSA300_0203		0.5	0.03074	putative lipoprotein
SAUSA300_0358		0.5	0.00000	putative methyltransferase
SAUSA300_1226		0.5	0.00000	homoserine dehydrogenase
SAUSA300_1288	*dapA*	0.5	0.00000	dihydrodipicolinate synthase
SAUSA300_1290	*dapD*	0.5	0.00000	tetrahydrodipicolinate acetyltransferase
SAUSA300_2555		0.5	0.00400	glutathione peroxidase
SAUSA300_1287	*asd*	0.5	0.00000	aspartate semialdehyde dehydrogenase
SAUSA300_1228	*thrB*	0.5	0.00000	homoserine kinase
SAUSA300_2006	*ilvD*	0.4	0.04247	dihydroxy-acid dehydratase
SAUSA300_0202		0.4	0.02930	peptide ABC transporter permease protein
SAUSA300_1225		0.4	0.00022	aspartate kinase
SAUSA300_1289	*dapB*	0.4	0.00000	dihydrodipicolinate reductase
SAUSA300_0175		0.4	0.00000	putative lipoprotein
SAUSA300_0174		0.4	0.00000	conserved hypothetical protein
SAUSA300_0359		0.4	0.00000	trans-sulfuration enzyme family protein
SAUSA300_0436		0.4	0.00084	ABC transporter permease protein
SAUSA300_0807		0.4	0.00073	conserved hypothetical protein
SAUSA300_2361		0.4	0.00000	conserved hypothetical protein
SAUSA300_0176		0.4	0.00000	ABC transporter permease protein
SAUSA300_0357	*metE*	0.4	0.00000	5-methyltetrahydropteroyltriglutamate-homocysteine S-methyltransferase
SAUSA300_2413		0.4	0.02241	conserved hypothetical protein
SAUSA300_2414		0.4	0.00905	conserved hypothetical protein
SAUSA300_0806		0.4	0.00562	conserved hypothetical protein
SAUSA300_0804		0.4	0.00022	putative transcriptional regulator
SAUSA300_0055		0.3	0.00000	alcohol dehydrogenase zinc-containing
SAUSA300_0950	*sspB*	0.3	0.00000	cysteine protease precursor
SAUSA300_0949	*sspC*	0.3	0.00000	cysteine protease
SAUSA300_1903		0.3	0.00016	conserved hypothetical protein
SAUSA300_0435		0.3	0.00000	ABC transporter ATP-binding protein
SAUSA300_0928	*comK*	0.3	0.00000	competence transcription factor
SAUSA300_0805		0.3	0.00000	pathogenicity island protein
SAUSA300_0201		0.3	0.00009	peptide ABC transporter permease protein
SAUSA300_0951	*sspA*	0.2	0.00000	V8 protease

SAUSA300_2052	0.1	0.00000	single-stranded DNA- binding protein family
SAUSA300_0489	0.0	0.00000	putative cell division protein FtsH

Supplementary Table 6. The USA300 genes affected by the *ftsH* deletion at both RNA and protein levels.

Up-regulated

Gene ID	Name	*ftsH*/WT		*p* value[a]	Product
		RNA	Protein		
0170		2.1	1.9	0.000	aldehyde dehydrogenase
0352		1.4	3.0	0.030	ABC transporter ATP-binding protein
0490	*hslO*	7.2	6.9	0.000	Hsp33-like chaperonin
1296		1.5	3.2	0.028	conserved hypothetical protein
1344		1.5	2.0	0.021	putative DNA replication protein DnaD
1913		1.4	2.2	0.048	ABC transporter ATP-binding protein
2306	*hrtA*	127.0	55.3	0.000	ABC transporter ATP-binding protein
2307	*hrtB*	211.0	26.8	0.000	ABC transporter permease protein
2367	*hlgB*	1.5	3.4	0.007	gamma-hemolysin component B
2390	*opuCd*	1.5	6.4	0.025	glycine betaine/carnitine/choline transport system permease
2459		1.9	1.7	0.007	transcriptional regulator MarR family
2494		1.8	1.8	0.000	copper-translocating P-type ATPase
2565	*clfB*	1.5	1.8	0.001	clumping factor B
2603	*lip*	1.4	2.4	0.047	triacylglycerol lipase precursor
2637		14.2	5.3	0.000	conserved hypothetical protein
2640		365.9	130.7	0.000	putative transcriptional regulator
2643		1.5	1.8	0.021	putative chromosome partioning protein ParB family

Down-regulated

Gene ID	Name	RNA	Protein	*p* value[a]	Product
0173		0.6	0.6	0.000	conserved hypothetical protein
0435		0.3	0.5	0.000	ABC transporter ATP-binding protein
0489		0.0	0.0	0.000	putative cell division protein FtsH
0969	*purS*	0.5	0.6	0.043	phosphoribosylformylglycinamidine synthase
0971	*purL*	0.6	0.6	0.025	phosphoribosylformylglycinamidine synthase II
0976	*purD*	0.7	0.6	0.040	phosphoribosylamine--glycine ligase
2225	*moaC*	0.7	0.7	0.010	molybdenum cofactor biosynthesis protein C
2276		0.7	0.7	0.007	peptidase M20/M25/M40 family
2331		0.6	0.6	0.001	transcriptional regulator MarR family

[a] For RNA-seq data.

Supplementary Table 7. Bacterial strains and plasmids used in this study

Strain or plasmid	Relevant characteristic	Origin or reference
E. coli		
DH5α	Plasmid free, restriction deficient	New England Biolabs
S. aureus		
RN4220	Restriction deficient, prophage cured	[1]
Newman	Clinical isolate, L18P substitution in SaeS	[2]
NMΔ*sae*	Newman with deletion of the *sae* operon	[3]
NMΔ*ftsH*	Newman with deletion of the *ftsH*	This study
USA300-P23	USA300-0114 without plasmid 2 and 3	[4]
USA300-P23Δ*ftsH*	USA300-0114 with deletion of the *ftsH*	This study
ΦNΞ-12830	Transposon mutant of *coa*, Phoenix library	[5]
ΦNΞ-09659	Transposon mutant of *eap*, Phoenix library	[5]
Plasmid		
pKOR1	Allelic replacement plasmid	[6]
pKOR1Δ*ftsH*	pKOR1 containing *ftsH* deletion cassette	This study
pCL55	An integration vector for *S. aureus*	[7]
pYJ335	An *E.coli*- *S. aureus* shuttle vector	[8]
p*ftsH*	pCL55 carrying the *ftsH* gene with His-tag sequence at the C-terminus	This study

p*ftsH* ^{K211N}	p*ftsH* carrying K211N mutation	This study
p*ftsH* ^{H431A}	p*ftsH* carrying H431A mutation	This study
pYJ-MurG-His_6	pYJ335 carrying *murG* with His_6-tag sequence	This study
pYJ- CydA-His_6	pYJ335 carrying *cydA* with His_6-tag sequence	This study
pYJ-Ffh-His_6	pYJ335 carrying *ffh* with His_6-tag sequence	This study
pYJ-HemA-His_6	pYJ335 carrying *hemA* with His_6-tag sequence	This study
pYJ-HrtB-His_6	pYJ335 carrying *hrtB* with His_6-tag sequence	This study
pYJ-SaeQ-His_6	pYJ335 carrying *saeQ* with His_6-tag sequence	This study
pYJ-FtsH	pYJ335 carrying *ftsH*	This study
pYJ-SrtA-His_6	pYJ335 carrying *srtA* with His_6-tag sequence	This study
pCL-FtsH ^{H431A}-Flag	pCL55 producing Flag-tagged $FtsH^{H431A}$	This study
pCL-RSusa-Flag	pCL55 producing SaeR and flag-tagged SaeS	[9]
pEap	pYJ335 carrying the *eap* gene with its own promote r	This study
pCL-RS^L	pCL55 carrying *saeRS* from USA300	[4]
pCL-RS^P	pCL55 carrying *saeRS* from Newman	[4]

Supplementary Table 8. Oligonucleotides used in this study

Name	Sequence (5’ → 3’)	Target
P236	ATTGGAAGTGGATAACGGTACCGGTTCCGAGGCTC	LIC for pKOR1
P237	ATTGGATTGGAAGTACGGGCCCGAGCTTAAGACTGG	LIC for pKOR1
P83	TTATCCACTTCCAATGCGATTAGAGATACATTCTAATTTAC	For *ftsH* deletion
P84	GCGTCATTTCCTCCTACTTCC	For *ftsH* deletion
P85	GGAAGTAGGAGGAAATGACGCTCGATTATATTCAGTACCTCTTTC	For *ftsH* deletion
P86	TACTTCCAATCCAATG CCACAGAAATGACATACTGCTTC	For *ftsH* deletion
P35	ATTGGAAGTGGATAACCGGAGGAGGGATGTAAAATGTGG	LIC for pCL55
P80	ATTGGATTGGAAGTACGAATTCTTGAAGACGAAAGGGCCTCG	LIC for pCL55
P2507	TTATCCACTTCCAATG CCCATTTATCAATTGATTTCATGG	p*ftsH*
P2508	TACTTCCAATCCAATGCTAATGATGATGATGATGATGTTTATTGTCTG<br>GGTGATTTGG	p*ftsH*
PL47	TACTTCCAATCCAATGTTACTTATCGTCGTCATCCTTGTAATC<br>TTTATTGTCTGGGTGATTTGG	pCL55-FtsH-Flag
P2576	CCTCCAGGTACTGGTAACACATTACTTGCTAGAG	p*ftsH* K211N
P2577	CTCTAGCAAGTAATGTGTTACCAGTACCTGGAGG	p*ftsH* K211N
P2578	CATCACGAAGCTGGTGCAACAATTATCGGTATG	p*ftsH* H431A
P2579	CATACCGATAATTGTTGCACCAGCTTCGTGATG	p*ftsH* H431A
P319	GAGCTCGAGCAGTAAGTGCACCAACTAATAA	For *eap* overexpression
P320	GAGGGTACCTTATTTATTTTTTTTGATTTAGTG	For *eap* overexpression
P321	GCAAACAGTATTTGTTAACCC	Real-time PCR for *clfB*
P322	GGATCTGCATAGTAGCTATCTG	Real-time PCR for *clfB*
P323	CAAGGTGCTGGTAAAACAACAAC	Real-time PCR for *ffh*
P324	CTTGATCTCCTTCACTGTATAC	Real-time PCR for *ffh*
P325	CATAACAAAGGCTCCAAAGAAAC	Real-time PCR for *saeQ*
P326	CACCAGAGTGGTATAAGTGG	Real-time PCR for *saeQ*

P327	GACGGTCAAAGCAAGAAATTC	Real-time PCR for *htrA*
P328	GATTGTTTGTAACGATATAAGCTG	Real-time PCR for *htrA*
P329	GACATTAAACCAGCACCAATC	Real-time PCR for *1351*
P330	GCCATTCAAGATCACCAAGG	Real-time PCR for *1351*
P331	GAGACGCTGCGGGAGGTCTAG	Real-time PCR for *pfoR*
P332	TAAGTCGACACCATCCGGCAC	Real-time PCR for *pfoR*
P333	GGTGTGGCCTTCCTCTATTTAAC	Real-time PCR for *lrgB*
P334	TACCTCCGATGATACGATGCC	Real-time PCR for *lrgB*
P335	GGGAAAGCGTATACGTCAGC	Real-time PCR for *0579*
P336	GTAATTTGTTGACTTAATGATC	Real-time PCR for *0579*
P339	GCAATTGACACTCGTAGGTC	Real-time PCR for *hrtA*
P340	CGCAACACGTTGCTTTTCAC	Real-time PCR for *hrtA*
P341	GCATTTTTCTATGTTATGACGATTC	Real-time PCR for *hrtB*
P342	CCGGCATCATAAATGATAGTC	Real-time PCR for *hrtB*
P343	CAGCTATGATGGGCGCAATG	Real-time PCR for *hslO*
P344	TACCGCTCGTCTTACATCAAG	Real-time PCR for *hslO*
P345	GATGCAAACTTACGTGAGCGAC	Real-time PCR for *hemA*
P346	CGATCAATACTGTCCATCGTT	Real-time PCR for *hemA*
P347	GATGATTTAGTAAAAGGGACC	Real-time PCR for *2640*
P348	GATTGCCACCGTTACACCCC	Real-time PCR for *2640*
P357	GTATGGCGAAGCACAGTGATC	Real-time PCR for *2637*
P358	GGTTTAGTGGAGCCATTTATTG	Real-time PCR for *2637*
P71	CTACGGCTGGTAAAGCTGAAG	Real-time PCR for *coa*
P72	TGAGCTACCTTCAAGACCTTC	Real-time PCR for *coa*
P250	CAGTACTGAAGCATGATAGAG	Real-time PCR for *eap*
P251	CTCTTAACATCTTTCGCATGAAC	Real-time PCR for *eap*
P312	CACGCTAAAGCATCAGTGACAG	Real-time PCR for *emp*
P313	CTAGCTGCCGATGAATCTGC	Real-time PCR for *emp*

P43	CAAATGATCACAGCATTTGGTACAG	Real-time PCR for *gyrB*
P44	CGGCATCAGTCATAATGACGAT	Real-time PCR for *gyrB*
P44	CGGCATCAGTCATAATGACGAT	Real-time PCR for *gyrB*
P371	ATTGGATTGGAAGTAC GATTACAAGGATGACGACGATAAG	LIC for pYJ335
P372	ATTGGAAGTGGATAAC AAGCTTATTT TAATTATACT CTATC	LIC for pYJ335
P39	TTATCCACTTCCAATG CCCATTTATCAATTGATTTCATGG	For *ftsH* overexpression
P370	TACTTCCAATCCAATG TTTATTGTCTGGGTGATTTGG	For *ftsH* overexpression
P484	GAGGAATTCCGTTTATTGTCTGGGTGATTTGGATC	For FtsHc production
PL111	GAGGGATCCATGAAAGCAAAAATGTACGATAATAATAAAC	For FtsHc production
P592	GAGGATATCGGATTTAGTTATGCAAAGTAATG	SaeQ expression (Sa)
P593	GAGGGTACCCTAATGATGATGATGATGTCCACGATCAGTAAGTGGGT C	SaeQ expression (Sa)
P570	GAG GATATC CTGAACCGCTCCTTTTTCTAC	MurG expression (Sa)
P571	GAGGGTACCCTAATGATGATGATGATGATGTTTAACGAAGAATCTTG CATATAAAG	MurG expression (Sa)
PL72	GAG GATATC CGGAGTTGAAAAAGCATCAAAATC	CydA expression (Sa)
PL73	GAGGGTACCCTAATGATGATGATGTTTTTCTATTTCTCCTCCTTGCTT C	CydA expression (Sa)
P574	GAG GATATC GATAATATAAGAAGAACTGGCG	Ffh expression (Sa)
P575	GAGGGTACCCTAATGATGATGATGATGATGAAACGGTAAATTCATAC CTTTTAAC	Ffh expression (Sa)
P576	GAG CCATGG GATACAATTGTAAGTTATTCATCAATTC	HemA expression (Sa)
P577	GAGGGTACCCTAATGATGATGATGATGATGTTCAAAACTAAAGATAC GTCGCGC	HemA expression (Sa)
PL74	GAG GATATC GTCACTAAGTGCATCCCGCGCTG	HrtB expression (Sa)
PL75	GAGGGTACCCTAATGATGATGATGTTCTGCACCTCCAATTGCTTCGA TAG	HrtB expression (Sa)
P601	GAG GGATCC ATGGTTACTCAAAATAAAAAG	MurG expression (Ec)[b]
P602	GAG GAATTC CGTTTAACGAAGAATCTTGCATATAAAG	MurG expression (Ec)

P603	GAG GGATCC ATGGCATTTGAAGGGTTATCAG	Ffh expression (Ec)
P604	GAG GAATTC CGAAACGGTAAATTCATACCTTTTAAC	Ffh expression (Ec)
P605	GAG GGATCC ATGCATTTTATTGCAATTAGTATAAATC	HemA expression (Ec)
P606	GAG GAATTC CG TTCAAAACTAAAGATACGTCGCGC	HemA expression (Ec)

[a] *S. aureus*; [b] *E. coli*

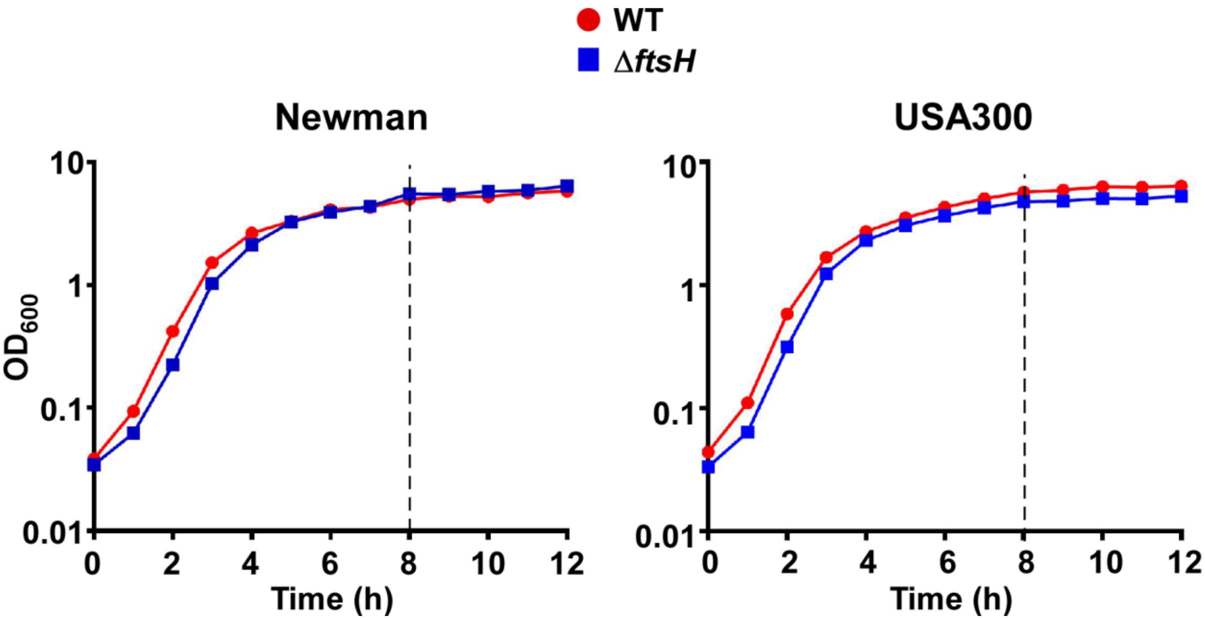

Supplementary Fig. 1. Grow curves for the test strains. The dotted lines indicate the time points when bacterial cells were collected for proteome analysis.

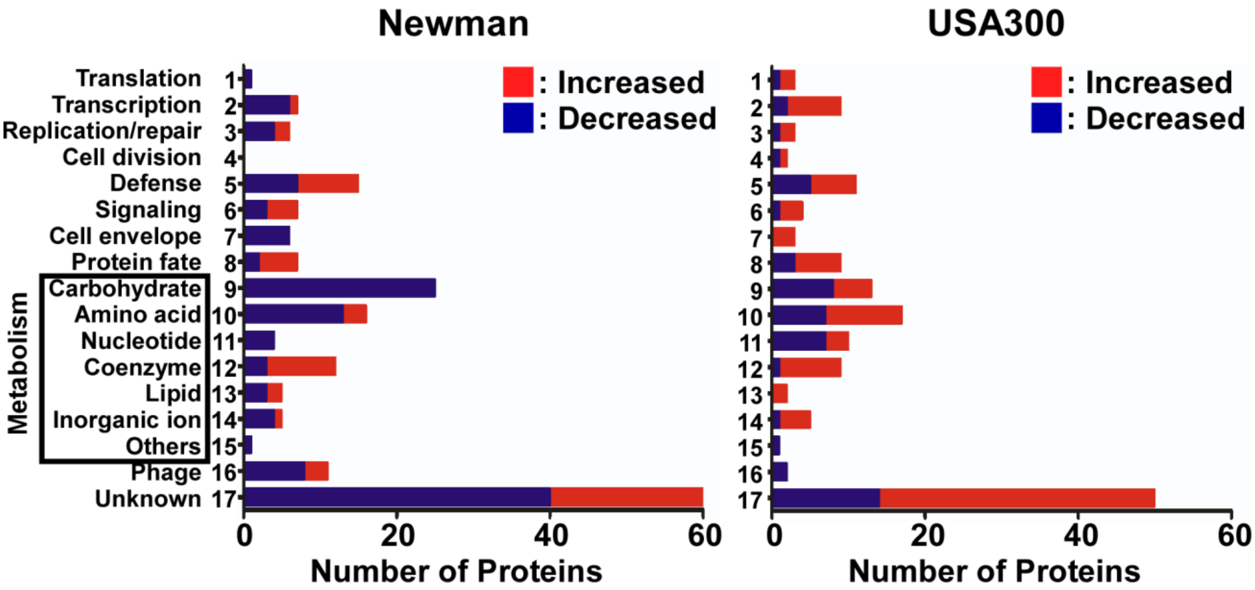

Supplementary Fig. 2. Functional classification of the proteins whose abundance was altered by *ftsH*-deletion.

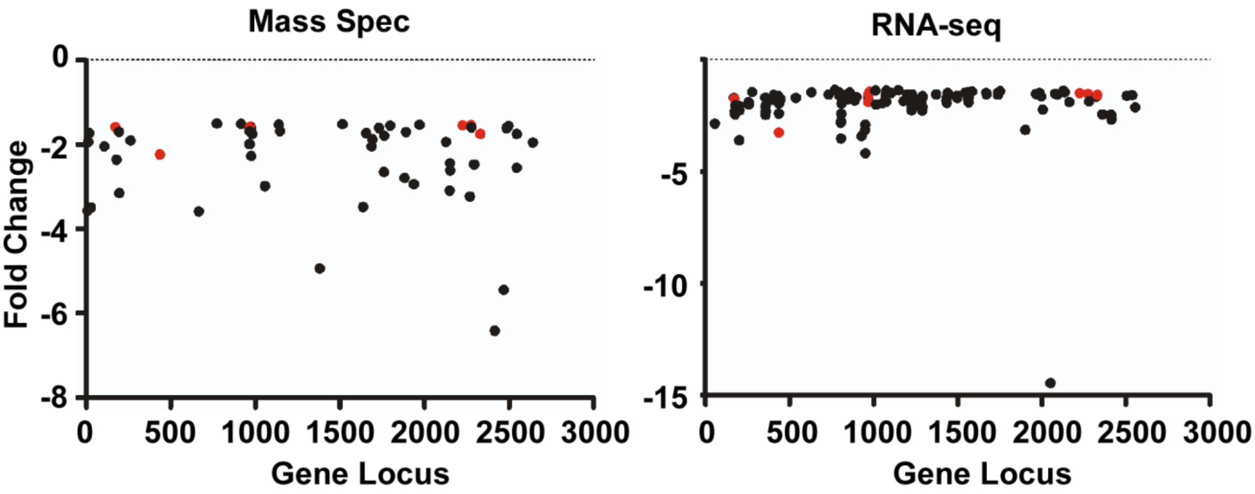

Supplementary Fig. 3. Comparison of the proteome (left panel) and transcriptome (right panel) analyses results for down-regulated proteins and genes. Red dots indicate the nine down-regulated proteins/genes shown in Fig. 3A. For clarity, the FtsH protein and the *ftsH* gene are omitted.

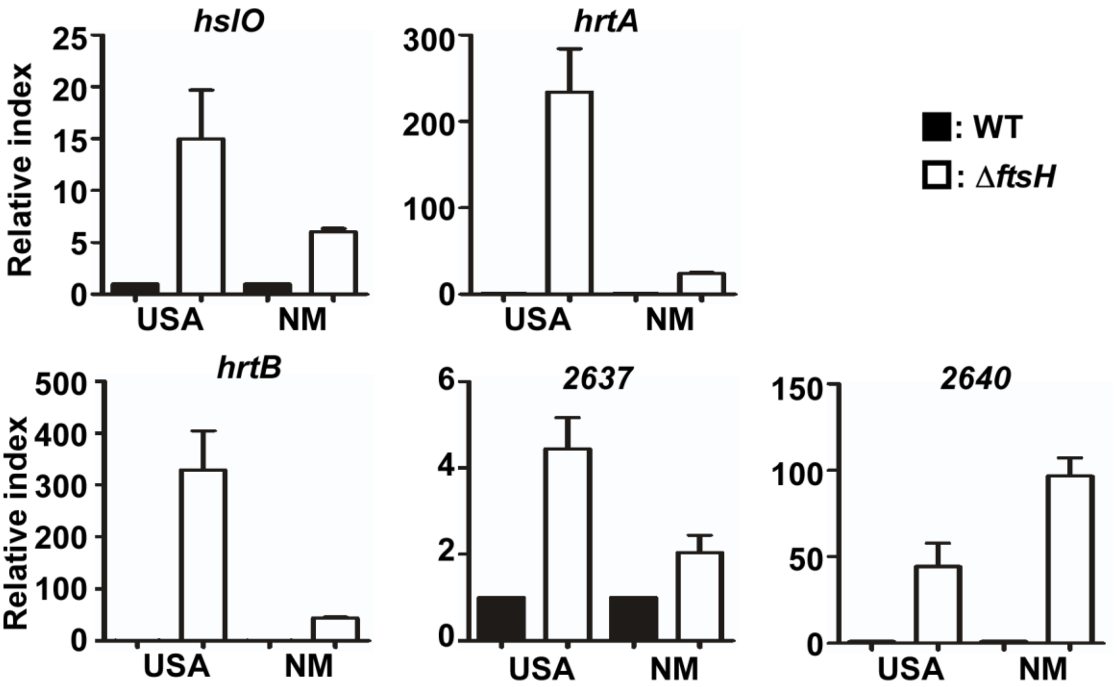

Supplementary Fig. 4. qRT-PCR analysis of the five genes whose transcription was increased in the *ftsH* mutant. Cells were grown in the same conditions as for the mass spectrometry analysis. Gene name or gene identifier of the genome of USA300_FPR3757 is shown above each graph. The assays were carried out in triplicate and repeated, resulting in similar results. Statistical significance was assessed by two-tailed unpaired Student's t-test. **, $p \leq 0.01$; ***, $p \leq 0.001$; ns, not significant. WT, wild type; *ΔftsH*; *ftsH*-deletion mutant; USA, USA300; NM, Newman.

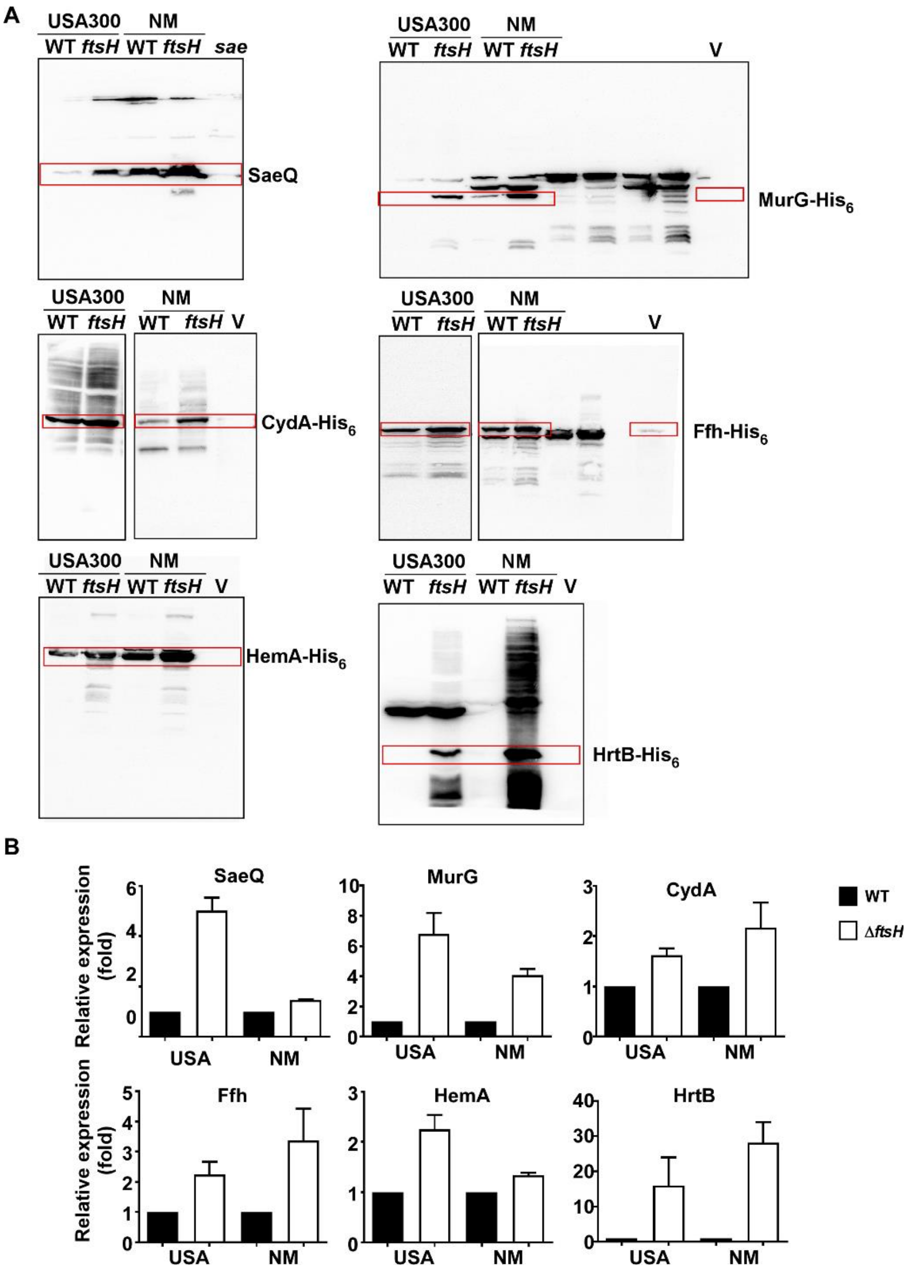

Supplementary Fig. 5. The full-length blots (A) and Quantification (B) of the Western blot analysis results shown in Fig. 4A. The cropped regions are indicated by red boxes. The blot for SaeQ was not included because of its unavailability. The blot results were quantified by densitometry analysis of two independent experiments. The error bars indicate standard deviations.

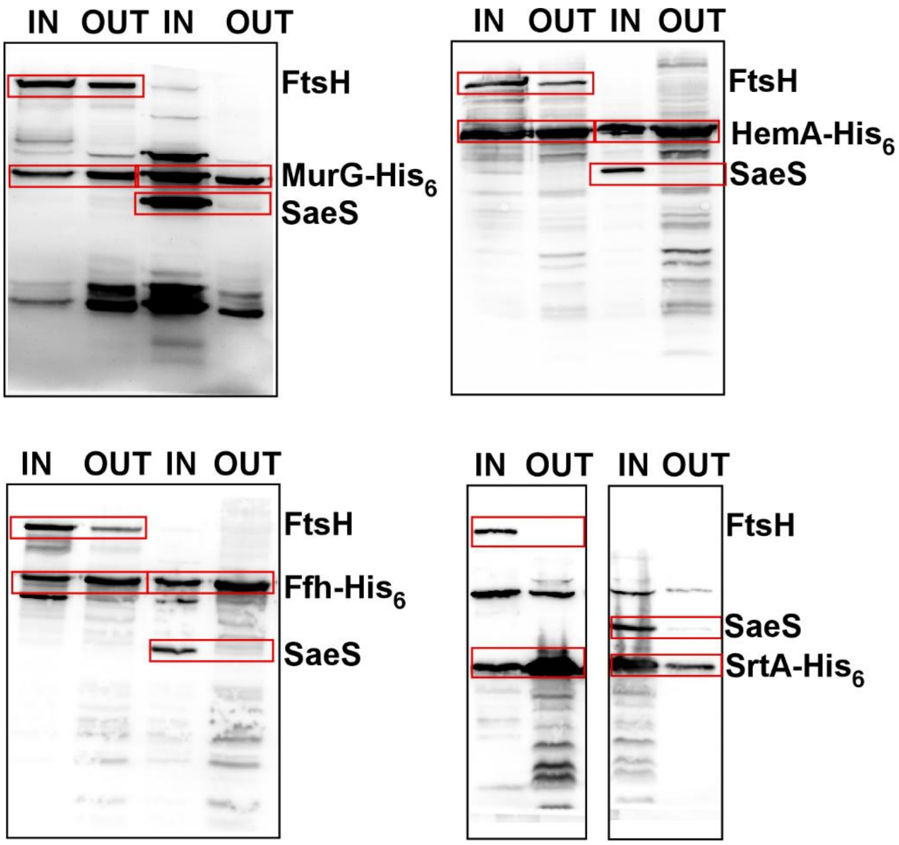

Supplementary Fig. 6. The full-length blots of the Western blot analysis results shown in Fig. 4B. The cropped regions are indicated by red boxes.

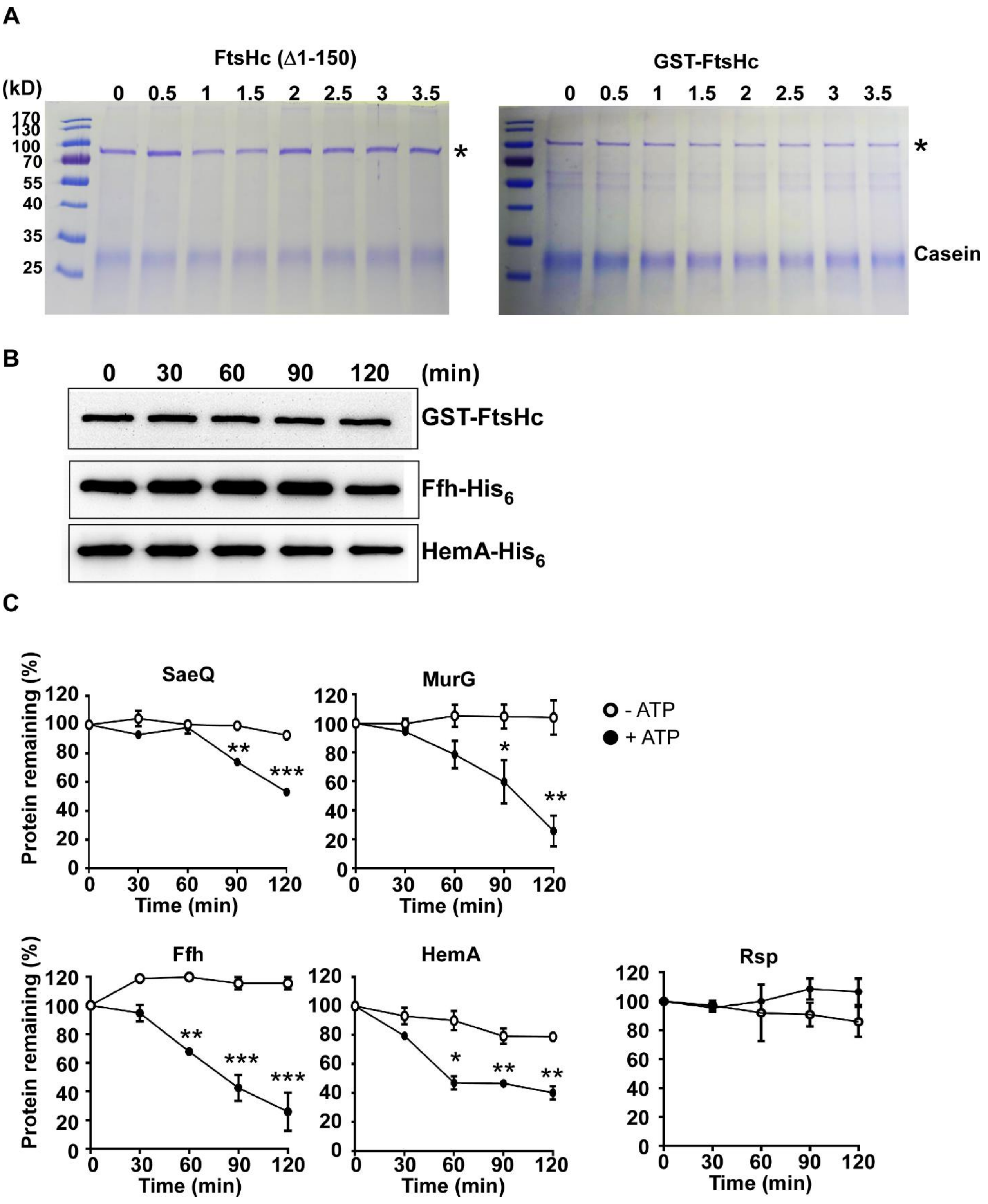
A
FtsHc (Δ1-150)
(kD)
0 0.5 1 1.5 2 2.5 3 3.5
170
130
100
70
55
40
35
25
*
GST-FtsHc
0 0.5 1 1.5 2 2.5 3 3.5
*
Casein
B
0 30 60 90 120 (min)
GST-FtsHc
Ffh-His6
HemA-His6
C
Protein remaining (%)
SaeQ
MurG
- ATP
+ ATP
Time (min)
Ffh
HemA
Rsp
Time (min)

Supplementary Fig. 7. Protein degradation assay for FtsH substrate proteins. (A) No or low proteolytic activity of FtsHc and GST-FtsHc. The proteolytic activities of FtsHc (left) and GST-FtsHc (right) were measured by using casein as a substrate. The FtsH proteins (10 μg) and casein (30 μg) were mixed, incubated at 42°C for the time indicated, and subjected to 12% SDS-PAGE and Coomassie blue staining. The star symbol (*) indicates the FtsH proteins. **(B)** Low proteolytic activity of GST-FtsHc toward substrate proteins. GST-FtsHc (4 μg) and the substrate proteins (12 μg) were mixed in the presence of ATP and incubated 42°C for the time indicated. The degradation of substrate proteins was analyzed by SDS-PAGE followed by Western blot analysis with anti-FtsH antibody (for GST-FtsHc) or anti-His_6-tag antibody (for the substrate proteins). **(C)** The quantification of Fig. 4C results. The corresponding proteins are indicated on each graph. The experiments were repeated three times. Statistical significance was assessed by two-tailed unpaired Student's t-test. *, $p \leq 0.05$; **, $p \leq 0.01$; ***, $p \leq 0.001$.

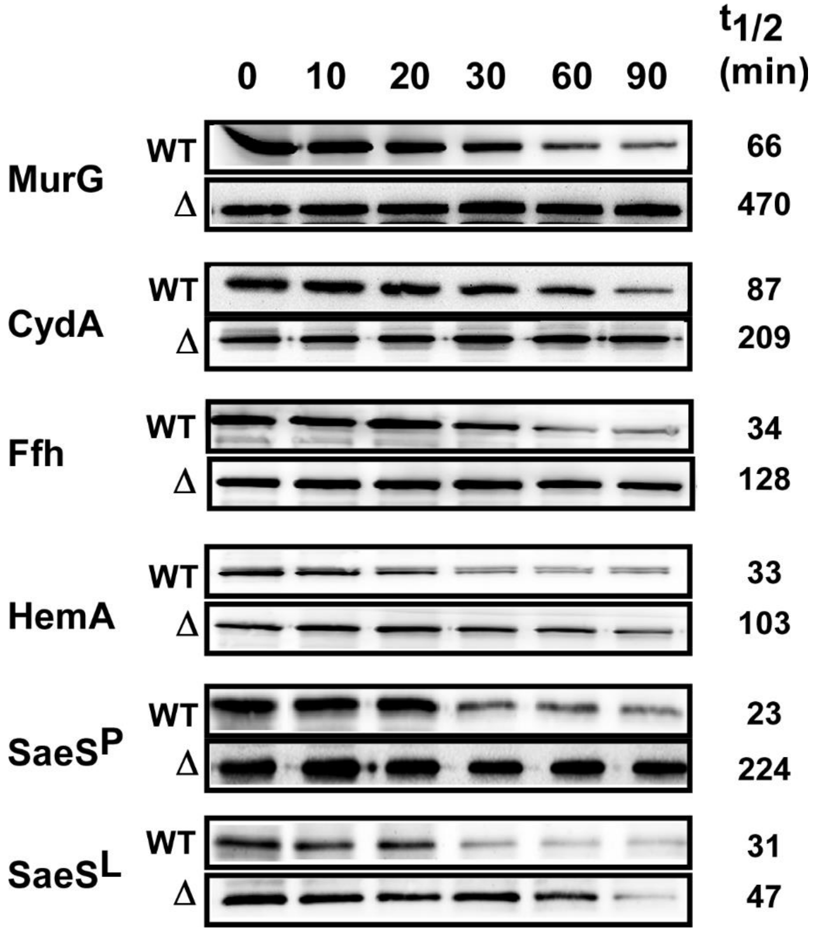

Supplementary Fig. 8. The effect of *ftsH* deletion on the stability of select FtsH substrate proteins. Test strains were treated with chloramphenicol (50 µg/mL); then, at the time points indicated, cells were collected, and the proteins were detected by Western blot analysis. $t_{1/2}$, half-life.

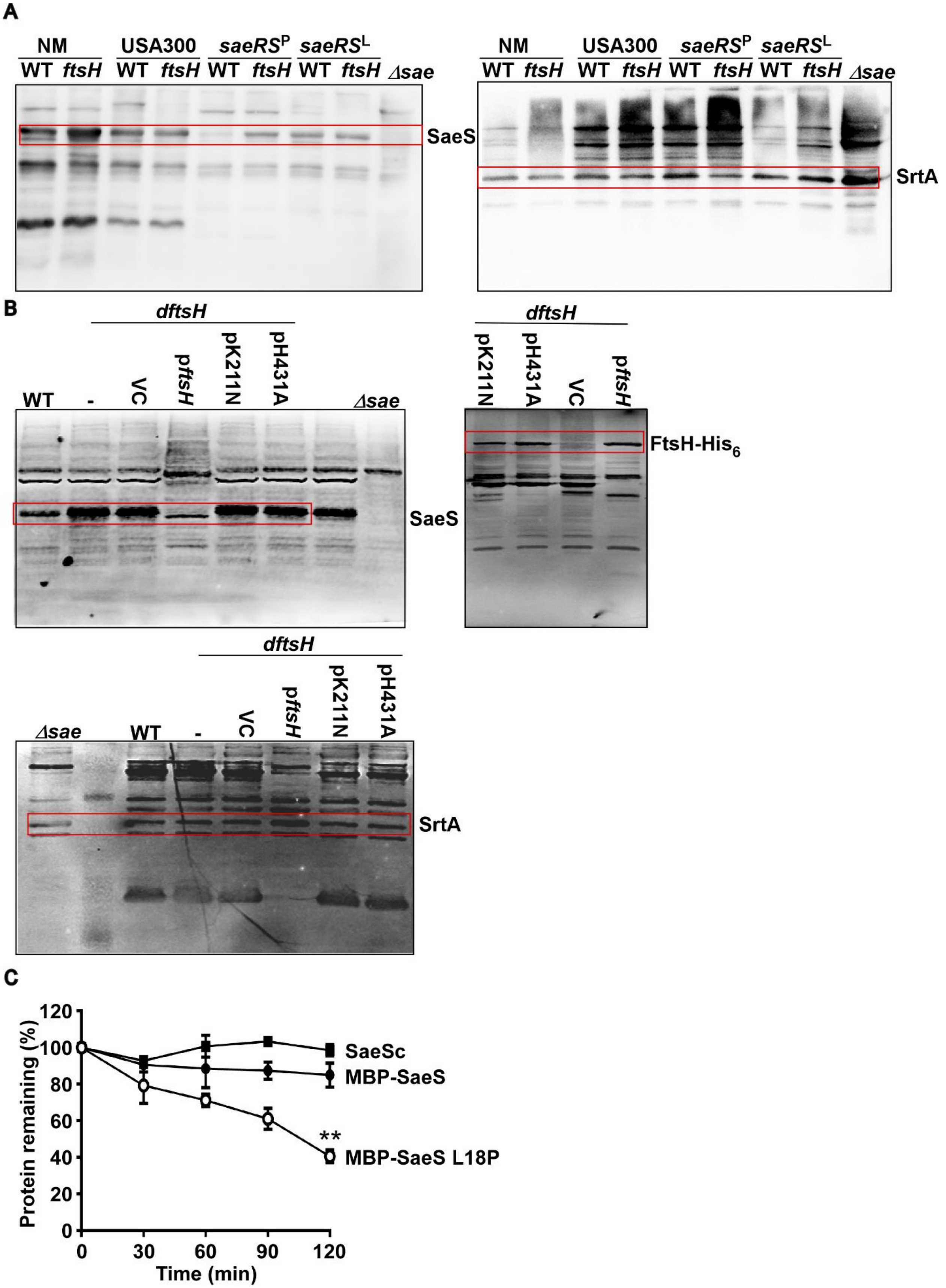
A
NM
USA300
saeRS^P
saeRS^L
WT ftsH WT ftsH WT ftsH WT ftsH Δsae
SaeS
SrtA
B
dftsH
WT - VC pftsH pK211N pH431A Δsae
SaeS
pK211N pH431A VC pftsH
FtsH-His6
Δsae WT - VC pftsH pK211N pH431A
SrtA
C
Protein remaining (%)
Time (min)
0 30 60 90 120
0 20 40 60 80 100 120
SaeSc
MBP-SaeS
MBP-SaeS L18P
**

Supplementary Fig. 9. The full-length blots of the Western blot analysis results shown in Fig. 5A & B (A-B) and the quantification of the proteolytic assay results shown in Fig. 5C (C). The cropped regions are indicated by red rectangles. The quantification results are from three independent experiments. Statistical significance was assessed by two-tailed unpaired Student's t-test. **, $p \leq 0.01$.

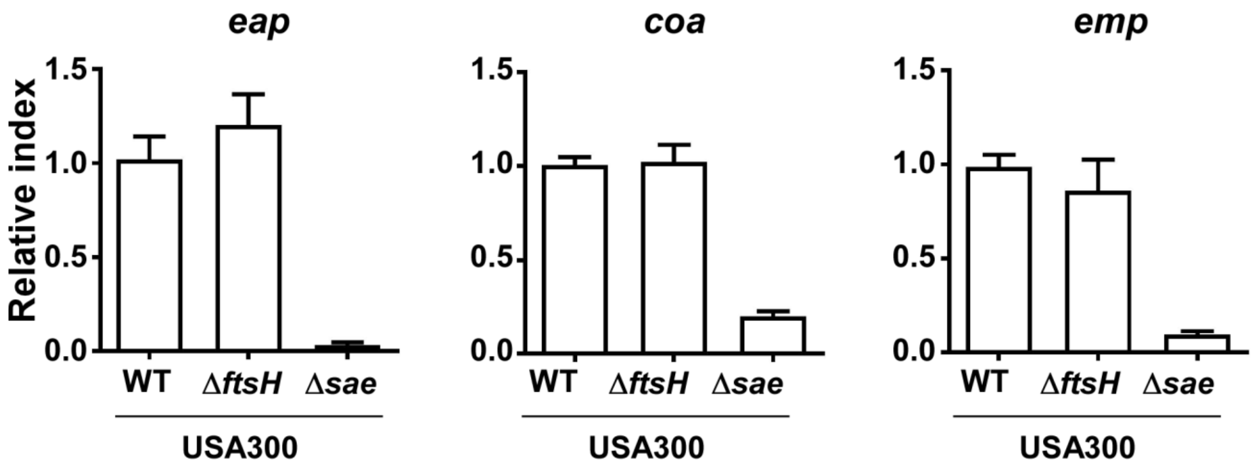

Supplementary Fig. 10. Effect of the *ftsH* deletion on the transcription of *eap, coa,* and *emp* in the USA300 strain background. The data are derived from three biological repeats. WT, wild type; Δ*ftsH,* the *ftsH*-deletion mutant; Δ*sae,* the *sae*-deletion mutant.

Methods

Protein half-life measurement

To examine the effect of FtsH on the stability of MurG, CydA, Ffh, and HemA, the Newman wild type or NMΔ*ftsH* strain carrying pYJ-MurG-His_6, pYJ-CydA-His_6, pYJ-Ffh-His_6, or pYJ-HemA-His_6 was grown in TSB containing anhydrotetracycline (100 ng/mL final concentration) with shaking at 37°C to exponential growth phase (OD_{600} = 0.5). To measure the half-life of SaeS, on the other hand, wild type and Δ*ftsH* strain of Newman and USA300 were grown in TSB to exponential growth phase (OD_{600} = 0.5). Then protein synthesis was stopped with chloramphenicol (50 µg/mL). At 0, 10, 20, 30, 60, 90 min post chloramphenicol treatment, cells, equivalent to 1 mL of OD600 = 0.5, were collected by centrifugation, and subjected to Western blot analysis with either His-tag antibody (for MurG, CydA, Ffh, and HemA) or SaeS antibody. The half-lives of the proteins were determined by densitometry analysis.

References

1. Kreiswirth, B. N. *et al.* The toxic shock syndrome exotoxin structural gene is not detectably transmitted by a prophage. *Nature* **305**, 709-712. (1983).
2. Duthie, E. S. & Lorenz, L. L. Staphylococcal coagulase; mode of action and antigenicity. *J Gen Microbiol* **6**, 95-107. (1952).
3. Sun, F. *et al.* Aureusimines in *Staphylococcus aureus* are not involved in virulence. *PLoS One* **5**, e15703, doi:10.1371/journal.pone.0015703 (2010).
4. Jeong, D. W. *et al.* Identification of P3 promoter and distinct roles of the two promoters of the SaeRS two-component system in Staphylococcus aureus. *J Bacteriol* **193**, 4672-4684 (2011).
5. Bae, T. *et al.* Staphylococcus aureus virulence genes identified by bursa aurealis mutagenesis and nematode killing. *Proc Natl Acad Sci U S A* **101**, 12312-12317 (2004).
6. Bae, T. & Schneewind, O. Allelic replacement in *Staphylococcus aureus* with inducible counter-selection. *Plasmid* **55**, 58-63 (2006).
7. Lee, C. Y., Buranen, S. L. & Ye, Z. H. Construction of single-copy integration vectors for *Staphylococcus aureus*. *Gene* **103**, 101-105 (1991).
8. Ji, Y., Marra, A., Rosenberg, M. & Woodnutt, G. Regulated antisense RNA eliminates alpha-toxin virulence in *Staphylococcus aureus* infection. *J Bacteriol* **181**, 6585-6590. (1999).

9 Liu, Q., Cho, H., Yeo, W. S. & Bae, T. The Extracytoplasmic Linker Peptide of the Sensor Protein SaeS Tunes the Kinase Activity Required for Staphylococcal Virulence in Response to Host Signals. *PLoS Pathog* **11**, e1004799 (2015).
